# Bayesian Computation Emerges in Generic Cortical Microcircuits through Spike-Timing-Dependent Plasticity

**DOI:** 10.1371/journal.pcbi.1003037

**Published:** 2013-04-25

**Authors:** Bernhard Nessler, Michael Pfeiffer, Lars Buesing, Wolfgang Maass

**Affiliations:** 1 Institute for Theoretical Computer Science, Graz University of Technology, Graz, Austria; 2 Institute of Neuroinformatics, University of Zürich and ETH Zürich, Zürich, Switzerland; Indiana University, United States of America

## Abstract

The principles by which networks of neurons compute, and how spike-timing dependent plasticity (STDP) of synaptic weights generates and maintains their computational function, are unknown. Preceding work has shown that soft winner-take-all (WTA) circuits, where pyramidal neurons inhibit each other via interneurons, are a common motif of cortical microcircuits. We show through theoretical analysis and computer simulations that Bayesian computation is induced in these network motifs through STDP in combination with activity-dependent changes in the excitability of neurons. The fundamental components of this emergent Bayesian computation are priors that result from adaptation of neuronal excitability and implicit generative models for hidden causes that are created in the synaptic weights through STDP. In fact, a surprising result is that STDP is able to approximate a powerful principle for fitting such implicit generative models to high-dimensional spike inputs: Expectation Maximization. Our results suggest that the experimentally observed spontaneous activity and trial-to-trial variability of cortical neurons are essential features of their information processing capability, since their functional role is to represent probability distributions rather than static neural codes. Furthermore it suggests networks of Bayesian computation modules as a new model for distributed information processing in the cortex.

## Author Summary

How do neurons learn to extract information from their inputs, and perform meaningful computations? Neurons receive inputs as continuous streams of action potentials or “spikes” that arrive at thousands of synapses. The strength of these synapses - the synaptic weight - undergoes constant modification. It has been demonstrated in numerous experiments that this modification depends on the temporal order of spikes in the pre- and postsynaptic neuron, a rule known as STDP, but it has remained unclear, how this contributes to higher level functions in neural network architectures. In this paper we show that STDP induces in a commonly found connectivity motif in the cortex - a winner-take-all (WTA) network - autonomous, self-organized learning of probabilistic models of the input. The resulting function of the neural circuit is Bayesian computation on the input spike trains. Such unsupervised learning has previously been studied extensively on an abstract, algorithmical level. We show that STDP approximates one of the most powerful learning methods in machine learning, Expectation-Maximization (EM). In a series of computer simulations we demonstrate that this enables STDP in WTA circuits to solve complex learning tasks, reaching a performance level that surpasses previous uses of spiking neural networks.

## Introduction

Numerous experimental data show that the brain applies principles of Bayesian inference for analyzing sensory stimuli, for reasoning and for producing adequate motor outputs 1–5. Bayesian inference has been suggested as a mechanism for the important task of probabilistic perception [6], in which hidden causes (e.g. the categories of objects) that explain noisy and potentially ambiguous sensory inputs have to be inferred. This process requires the combination of prior beliefs about the availability of causes in the environment, and probabilistic generative models of likely sensory observations that result from any given cause. By Bayes Theorem, the result of the inference process yields a *posterior* probability distribution over hidden causes that is computed by multiplying the *prior* probability with the *likelihood* of the sensory evidence for all possible causes. In this article we refer to the computation of posterior probabilities through a combination of probabilistic prior and likelihood models as Bayesian computation. It has previously been shown that priors and models that encode likelihoods of external stimuli for a given cause can be represented in the parameters of neural network models [6], [7]. However, in spite of the existing evidence that Bayesian computation is a primary information processing step in the brain, it has remained open how networks of neurons can acquire these priors and likelihood models, and how they combine them to arrive at posterior distributions of hidden causes.

The fundamental computational units of the brain, neurons and synapses, are well characterized. The synaptic connections are subject to various forms of plasticity, and recent experimental results have emphasized the role of STDP, which constantly modifies synaptic strengths (weights) in dependence of the difference between the firing times of the pre- and postsynaptic neurons (see [8], [9] for reviews). Functional consequences of STDP can resemble those of rate-based Hebbian models [10], but may also lead to the emergence of temporal coding [11] and rate-normalization [12], [13]. In addition, the excitability of neurons is modified through their firing activity [14]. Some hints about the organization of local computations in stereotypical columns or so-called cortical microcircuits [15] arises from data about the anatomical structure of these hypothesized basis computational modules of the brain. In particular, it has been observed that local ensembles of pyramidal neurons on layers 2/3 and layers 5/6 typically inhibit each other, via indirect synaptic connections involving inhibitory neurons [16]. These ubiquitous network motifs were called soft winner-take-all (WTA) circuits, and have been suggested as neural network models for implementing functions like non-linear selection [16], [17], normalization [18], selective attention [19], decision making [20], [21], or as primitives for general purpose computation [22], [23].

A comprehensive theory that explains the emergence of computational function in WTA networks of spiking neurons through STDP has so far been lacking. We show in this article that STDP and adaptations of neural excitability are likely to provide the fundamental components of Bayesian computation in soft WTA circuits, yielding representations of posterior distributions for hidden causes of high-dimensional spike inputs through the firing probabilities of pyramidal neurons. This is shown in detail for a simple, but very relevant feed-forward model of Bayesian inference, in which the distribution for a single hidden cause is inferred from the afferent spike trains. Our new theory thus describes how modules of soft WTA circuits can acquire and perform Bayesian computations to solve one of the fundamental tasks in perception, namely approximately inferring the category of an object from feed-forward input. Neural network models that can handle Bayesian inference in general graphical models, including bi-directional inference over arbitrary sets of random variables, explaining away effects, different statistical dependency models, or inference over time require more complex network architectures [24], [25], and are the topic of ongoing research. Such networks can be composed out of interconnected soft WTA circuits, which has been shown to be a powerful principle for designing neural networks that can solve arbitrary deterministic or stochastic computations [22], [23], [25]. Our theory can thus be seen as a first step towards learning the desired functionality of individual modules.

At the heart of this link between Bayesian computation and network motifs of cortical microcircuits lies a new theoretical insight on the micro-scale: If the STDP-induced changes in synaptic strength depend in a particular way on the current synaptic strength, STDP approximates for each synapse exponentially fast the conditional probability that the presynaptic neuron has fired just before the postsynaptic neuron (given that the postsynaptic neuron fires). This principle suggests that synaptic weights can be understood as conditional probabilities, and the ensemble of all weights of a neuron as a generative model for high-dimensional inputs that - after learning - causes it to fire with a probability that depends on how well its current input agrees with this generative model. The concept of a generative model is well known in theoretical neuroscience [26], [27], but it has so far primarily been applied in the context of an abstract non-spiking neural circuit architecture. In the Bayesian computations that we consider in this article, internal generative models are represented implicitly through the learned values of bottom-up weights in spiking soft-WTA circuits, and inference is carried out by neurons that integrate such synaptic inputs and compete for firing in a WTA circuit. In contrast to previous rate-based models for probabilistic inference [28]–[30] every spike in our model has a clear semantic interpretation: one spike indicates the instantaneous assignment of a certain value to an abstract variable represented by the firing neuron. In a Bayesian inference context, every input spike provides evidence for an observed variable, whereas every output spike represents one stochastic sample from the posterior distribution over hidden causes encoded in the circuit.

We show that STDP is able to approximate the arguably most powerful known learning principle for creating these implicit generative models in the synaptic weights: Expectation Maximization (EM). The fact that STDP approximates EM is remarkable, since it is known from machine learning that EM can solve a fundamental chicken-and-egg problem of unsupervised learning systems [31]: To detect - without a teacher - hidden causes for complex input data, and to induce separate learning agents to specialize each on one of the hidden causes. The problem is that as long as the hidden causes are unknown to the learning system, it cannot tell the hidden units what to specialize on. EM is an iterative process, where initial guesses of hidden causes are applied to the current input (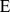-step) and successively improved (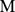-step), until a local maximum in the log-likelihood of the input data is reached. In fact, the basic idea of EM is so widely applicable and powerful that most state-of-the art machine learning approaches for discovering salient patterns or structures in real-world data without a human supervisor rely on some form of EM [32]. We show that in our spiking soft-WTA circuit each output spike can be viewed as an application of the 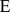-step of EM. The subsequent modification of the synaptic weights between the presynaptic input neurons and the very neuron that has fired the postsynaptic spike according to STDP can be viewed as a move in the direction of the 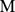-step of a stochastic online EM procedure. This procedure strives to create optimal internal models for high-dimensional spike inputs by maximizing their 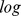-likelihood. We refer to this interpretation of the functional role of STDP in the context of spiking WTA circuits as **s**pike-based **E**xpectation **M**aximization (SEM).

This analysis gives rise to a new perspective of the computational role of local WTA circuits as parts of cortical microcircuits, and the role of STDP in such circuits: The fundamental computational operations of Bayesian computation (Bayes Theorem) for the inference of hidden causes from bottom-up input emerge in these local circuits through plasticity. The pyramidal neurons in the WTA circuit encode in their spikes samples from a posterior distribution over hidden causes for high-dimensional spike inputs. Inhibition in the WTA accounts for normalization [18], and in addition controls the rate at which samples are generated. The necessary multiplication of likelihoods (given by implicit generative models that are learned and encoded in their synaptic weights) with simultaneously learned priors for hidden causes (in our model encoded in the neuronal excitability), does not require any extra computational machinery. Instead, it is automatically carried out (on the 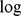 scale) through linear features of standard neuron models. We demonstrate the emergent computational capability of these self-organizing modules for Bayesian computation through computer simulations. In fact, it turns out that a resulting configuration of networks of spiking neurons can solve demanding computational tasks, such as the discovery of prototypes for handwritten digits without any supervision. We also show that these emergent Bayesian computation modules are able to discover, and communicate through a sparse output spike code, repeating spatio-temporal patterns of input spikes. Since such self-adaptive computing and discrimination capability on high-dimensional spatio-temporal spike patterns is not only essential for early sensory processing, but could represent a generic information processing step also in higher cortical areas, our analysis suggests to consider networks of self-organizing modules for spike-based Bayesian computation as a new model for distributed real-time information processing in the brain.

Preliminary ideas for a spike-based implementation of EM were already presented in the extended abstract [20], where we analyzed the relationship of a simple STDP rule to a Hebbian learning rule, and sketched a proof for stochastic online EM. In the present work we provide a rigorous mathematical analysis of the learning procedure, a proof of convergence, expand the framework towards learning spatio-temporal spike patterns, and discuss in detail the relationship of our STDP rule to experimental results, as well as the interpretation of spikes as samples from instantaneous posterior probability distributions in the context of EM.

## Results

In this section we define a simple model circuit and show that every spiking event of the circuit can be described as one independent sample of a discrete probability distribution, which itself evolves over time in response to the spiking input. Within this network we analyze a variant of a STDP rule, in which the strength of potentiation depends on the current weight value. This local learning rule, which is supported by experimental data, and at intermediate spike frequencies closely resembles typical STDP rules from the literature, drives every synaptic weight to converge stochastically to the log of the probability that the presynaptic input neuron fired a spike within a short time window 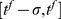, before the postsynaptic neuron spikes at time 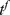:
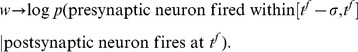
(1)We then show that the network model can be viewed as performing Bayesian computation, meaning that every spike can be understood as a sample from a posterior distribution over hidden causes in a generative probabilistic model, which combines prior probabilities and evidence from current input spike trains.

This understanding of spikes as samples of hidden causes leads to the central result of this paper. We show that STDP implements a stochastic version of Expectation Maximization for the unsupervised learning of the generative model and present convergence results for SEM. Importantly, this implementation of EM is based on spike events, rather than spike rates.

Finally we discuss how our model can be implemented with biologically realistic mechanisms. In particular this provides a link between mechanisms for lateral inhibition in WTA circuits and learning of probabilistic models. We finally demonstrate in several computer experiments that SEM can solve very demanding tasks, such as detecting and learning repeatedly occurring spike patterns, and learning models for images of handwritten digits without any supervision.

### Definition of the network model

**Figure 1:**
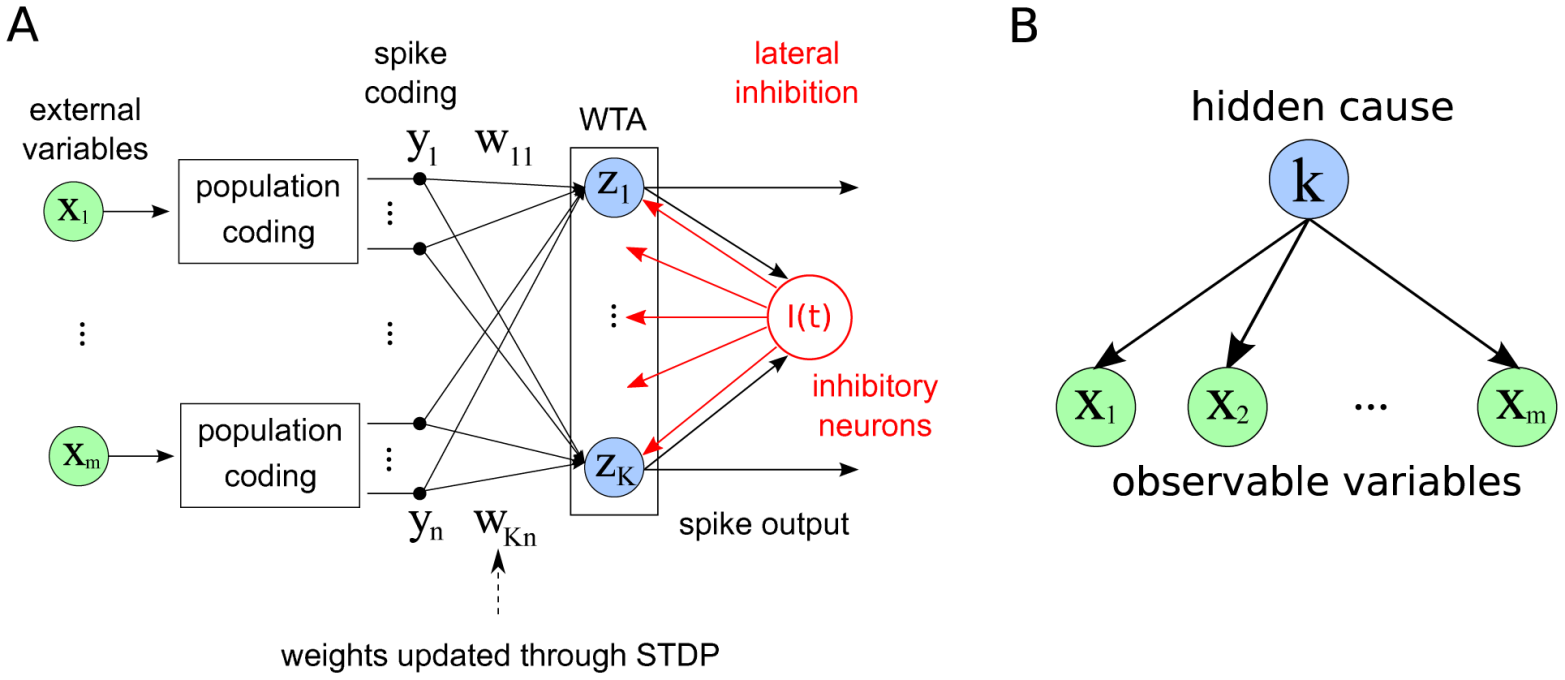
The network model and its probabilistic interpretation. **A** Circuit architecture. External input variables are encoded by populations of spiking neurons, which feed into a Winner-take-all (WTA) circuit. Neurons within the WTA circuit compete via lateral inhibition and have their input weights updated through STDP. Spikes from the WTA circuit constitute the output of the system. **B** Generative probabilistic model for a multinomial mixture: A vector of external input variables 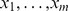 is dependent on a hidden cause, which is represented by the discrete random variable 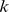. In this model it is assumed that the 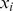
's are conditionally independent of each other, given 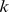. The inference task is to infer the value of 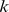, given the observations for 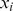. Our neuronal network model encodes the conditional probabilities of the graphical model into the weight vector 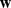, such that the activity of the network can be understood as execution of this inference task.

Our model consists of a network of spiking neurons, arranged in a WTA circuit, which is one of the most frequently studied connectivity patterns (or network motifs) of cortical microcircuits [16]. The input of the circuit is represented by the excitatory neurons 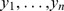. This input projects to a population of excitatory neurons 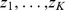 that are arranged in a WTA circuit (see Fig. 1). We model the effect of lateral inhibition, which is the competition mechanism of a WTA circuit [33], by a common inhibitory signal 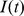 that is fed to all 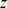 neurons and in turn depends on the activity of the 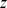 neurons. Evidence for such common local inhibitory signals for nearby neurons arises from numerous experimental results, see e.g. [16], [34]–[36]. We do not a priori impose a specific functional relationship between the common inhibition signal and the excitatory activity. Instead we will later derive necessary conditions for this relationship, and propose a mechanism that we use for the experiments.

The individual units 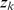 are modeled by a simplified Spike Response Model [37] in which the membrane potential is computed as the difference between the excitatory input 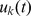 and the common inhibition term 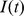. 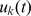 sums up the excitatory inputs from neurons 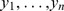 as
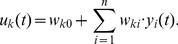
(2)

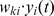
 models the EPSPs evoked by spikes of the presynaptic neuron 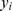, and 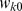 models the intrinsic excitability of the neuron 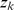. In order to simplify our analysis we assume that the EPSP can be modeled as a step function with amplitude 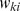, i.e., 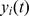 it takes on the value 1 in a finite time window of length 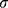 after a spike and is zero before and afterwards. Further spikes within this time window do not contribute additively to the EPSP, but only extend the time window during which the EPSP is in the high state. We will later show how to extend our results to the case of realistically shaped and additive EPSPs.

We use a stochastic firing model for 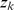, in which the firing probability depends exponentially on the membrane potential, i.e.,
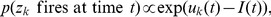
(3)which is in good agreement with most experimental data [38]. We can thus model the firing behavior of every neuron 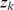 in the WTA as an independent inhomogeneous Poisson process whose instantaneous firing rate is given by 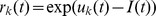.

In order to understand how this network model generates samples from a probability distribution, we first observe that the combined firing activity of the neurons 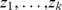 in the WTA circuit is simply the sum of the 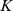 independent Poisson processes, and can thus again be modeled as an inhomogeneous Poisson process with rate 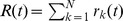. Furthermore, in any infinitesimally small time interval 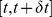, the neuron 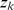 spikes with probability 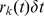. Thus, if we know that at some point in time 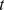, i.e. within 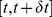, *one* of the neurons 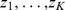 produces an output spike, the conditional probability 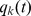 that this spike originated from neuron 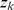 can be expressed as
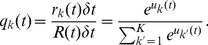
(4)Every single spike from the WTA circuit can thus be seen as an independent sample from the instantaneous distribution in Eq. (4) at the time of the spike. Although the instantaneous firing rate of every neuron directly depends on the value of the inhibition 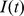, the relative proportion of the rate 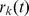 to the total WTA firing rate 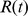 is independent of the inhibition, because all neurons receive the same inhibition signal 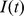. Note that 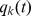 determines only the value of the sample at time 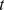, but not the time point at which a sample is created. The temporal structure of the sampling process depends only on the overall firing rate 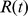.

This implementation of a stochastic WTA circuit does not constrain in any way the kind of spike patterns that can be produced. Every neuron fires independently according to a Poisson process, so it is perfectly possible (and sometimes desirable) that there are two or more neurons that fire (quasi) simultaneously. This is no contradiction to the above theoretical argument of single spikes as samples. There we assumed that there was only one spike at a time inside a time window, but since we assumed these windows to be infinitesimally small, the probability of two spikes occurring exactly at the same point in continuous time is zero.

#### Synaptic and intrinsic plasticity

**Figure 2:**
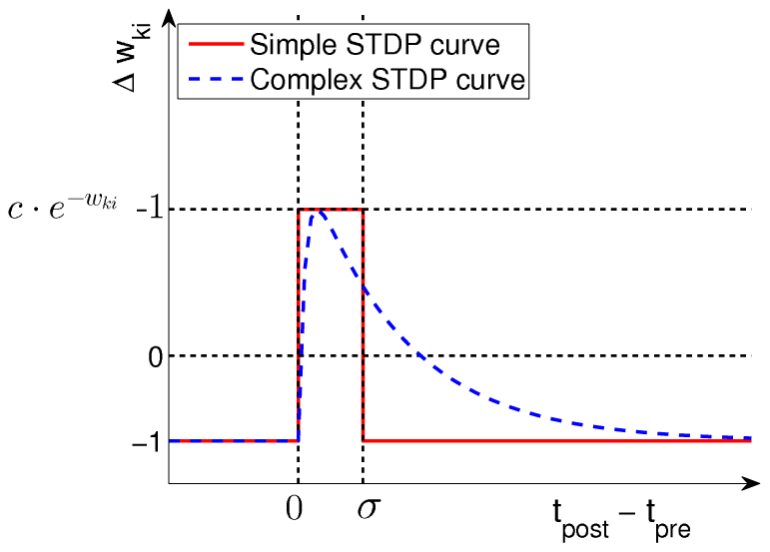
Learning curves for STDP. Under the simple STDP model (red curve), potentiation occurs only if the postsynaptic spike falls within a time window of length 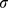 (typically 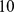
ms) after the presynaptic spike. The convergence properties of this simpler version in conjunction with rectangular non-additive EPSPs are easier to analyze. In our simulations we use the more complex version (blue dashed curve) in combination with EPSPs that are modeled as biologically realistic 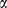-kernels (with plausible time-constants for rise and decay of 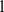 respectively 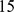 ms).

We can now establish a link between biologically plausible forms of spike-based learning in the above network model and learning via EM in probabilistic graphical models. The synaptic weights 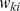 of excitatory connections between input neurons 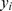 and neurons 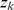 in the WTA circuit change due to STDP. Many different versions of STDP rules have emerged from experimental data [8], [39], [40]. For synaptic connections between excitatory neurons, most of them yield a long term potentiation (LTP) when the presynaptic neuron 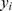 fires before the postsynaptic neuron 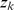, otherwise a long term depression (LTD). In our model we use a STDP rule in which the shape of the positive update follows the shape of EPSPs at the synapses, and in which the amplitude of the update 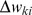 depends on the value of the synaptic weight 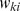 before the update as in Fig. 2. Specifically, we propose a rule in which the ratio of LTP and LTD amplitudes is inversely exponentially dependent on the current synaptic weight. LTP curves that mirror the EPSP shape are in accordance with previous studies, which analyzed optimal shapes of STDP curves under different mathematical criteria [41], [42]. The depression part of the rule in Fig. 2 is a flat offset that contrasts the potentiation. We will show later that this form of LTD occurs in our simulations only at very low repetition frequencies, and instead at natural frequencies our model gives rise to a form of STDP with spike-timing dependent LTD that is very similar to plasticity curves observed in biology [40], [43]. We will also analyze the relationship between this rule and a biologically more realistic STDP rule with an explicit time-decaying LTD part.

We can formulate this STDP-rule as a Hebbian learning rule 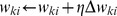 - with learning rate 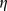 - which is triggered by a spike of the postsynaptic neuron 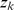 at time 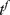. The dependence of 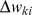 on the synaptic activity 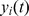 and the current value of the synaptic weight is given by
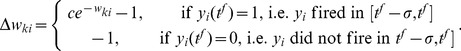
(5)Since 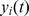 reflects the previously defined step function shape of the EPSP, this update rule is exactly equivalent to the simple STDP rule (solid red curve) in Fig. 2 for the case of the pairing of one pre- and one postsynaptic spike. The dependence on the presynaptic activity 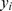 is reflected directly by the time difference 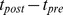 between the pre- and the postsynaptic spikes. According to this rule positive updates are only performed if the presynaptic neuron fired in a time window of 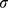 ms before the postsynaptic spike. This learning rule therefore respects the causality principle of LTP that is implied in Hebb's original formulation [44], rather than looking only at correlations of firing rates.

We can interpret the learning behavior of this simple STDP rule from a probabilistic perspective. Defining a stationary joint distribution 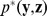 over the binary input activations 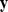 at the times of the postsynaptic spikes, and the binary vector 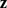, which indicates the source of the postsynaptic spike by setting one 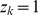, we show in Methods that the equilibrium condition of the expected update 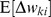 leads to the single solution
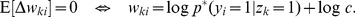
(6)This stochastic convergence to the log-probability of the presynaptic neuron being active right before the postsynaptic neuron fires is due to the exponential dependence of the potentiation term on the current weight value. Log-probabilities are necessarily negative values, whereas for biological neural networks we typically expect excitatory, i.e. positive weights from the excitatory input neurons. The parameter 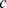 shifts the range of the values for the weights 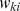 into the positive regime for 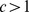. For the sake of simplicity we assume that 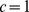 for the following theoretical analysis and we show in Methods that all results remain true for any positive value of 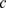.

In analogy to the plasticity of the synaptic weights we also explore a form of intrinsic plasticity of the neurons. We interpret 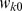 as an indicator for the excitability of the neuron 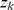 and apply a circuit-spike triggered update rule 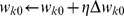 with
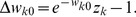
(7)Whenever a neuron 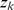 fires, the excitability is increased and the amount of increase is inversely exponentially dependent on the current excitability. Otherwise the excitability is decreased by a constant. Such positive feedback through use-dependent changes in the excitability of neurons were found in numerous experimental studies (see e.g. [14], [45]). This concrete model of intrinsic plasticity drives the excitability 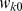 towards the only equilibrium point of the update rule, which is 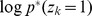. In Methods (see ‘Weight offsets and positive weights’) we show that the depression of the excitability can be modeled either as an effect of lateral inhibition from firing of neighboring neurons, or as a constant decay, independent of the instantaneous circuit activity. Both methods lead to different values 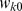, it is true, but encode identical instantaneous distributions 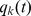.

Note, however, that also negative feedback effects on the excitability through homeostatic mechanisms were observed in experiments [13], [46]. In a forthcoming article [47] we show that the use of such homeostatic mechanisms instead of Eq. (7) in an, otherwise unchanged, network model may be interpreted as a posterior constraint in the context of EM.

#### Generative probabilistic model

The instantaneous spike distribution 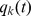 from Eq. (4) can be understood as the result of Bayesian inference in an underlying generative probabilistic model for the abstract multinomial observed variables 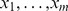 and a hidden cause 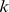. We define the probability distribution of the variables 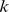 and 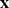, as shown by the graphical model in Fig. 1B, as 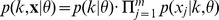. The parametrization 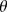 of the graphical model consists of a prior 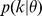
on 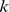, and conditional probabilities 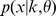 for every 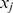.

The probabilistic model 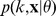 is a generative model and therefore serves two purposes: On the one hand, it can be used to generate samples of the hidden variable 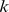 and the observable variables 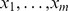. This is done by sampling 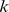 from the prior distribution, and then sampling the 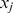
's, which depend on 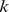 and can be generated according to the conditional probability tables. The resulting marginal distribution 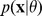 is a special case of a multinomial mixture distribution.

On the other hand, for any given observation of the vector 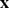, one can infer the value of the hidden cause 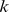 that led to the generation of this value for 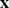. By application of Bayes' rule one can infer the posterior distribution 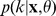 over all possible values of 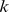, which is proportional to the product of the prior 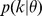 and the likelihood 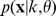.

We define population codes to represent the external observable variables 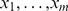 by the input neurons 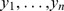, and the hidden variable 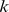 by the circuit neurons 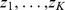: For every variable 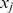 and every possible (discrete) value that 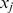 can adopt, there is exactly one neuron 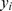 which represents this combination. We call 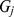 the set of the indices of all 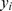
's that represent 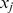, and we call 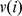 the possible value of 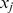 that is represented by neuron 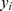. Thus we can define an interpretation for the spikes from the input neurons by
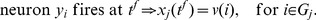
(8)A spike from the group 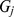 represents an instantaneous evidence about the observable variable 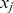 at the time of the spike. In the same way every neuron 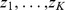 represents one of the 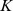 possible values for the hidden variable 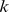, and every single spike conveys an instantaneous value for 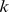. We can safely assume that all neurons - including the input neurons - fire according to their individual local stochastic processes or at least exhibit some local stochastic jitter. For the theoretical analysis one can regard a spike as an instantaneous event at a single point in time. Thus in a continuous time no two events from such local stochastic processes can happen at exactly the same point in time. Thus, there is never more than one spike at any single point in time within a group 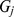, and every spike can be treated as a proper sample from 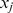. However, the neurons 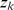 coding for hidden causes need to integrate evidence from multiple inputs, and thus need a mechanism to retain the instantaneous evidence from a single spike over time, in order to learn from spatial and temporal correlations in the input.

In our framework this is modeled by postsynaptic potentials on the side of the receiving neurons that are generated in response to input spikes, and, by their shape, represent evidence over time. In the simple case of the non-additive step-function model of the EPSP in Eq. (2), every spike indicates new evidence for the encoded variable that remains valid during a time window of 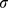, after which the evidence is cleared. In the case that there is no spike from one group 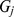 within a time window of length 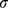, this is interpreted as missing evidence (or missing value) for 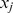 in a subsequent inference. In practice it may also occur that EPSPs within a group 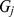 of input neurons overlap, which would indicate contradicting evidence for 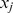. For the theoretical analysis we will first assume that spikes from different input neurons within the same group 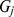 are not closer in time than 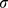, in order to avoid such conflicts. We will later drop this restriction in the extension to more realistically shaped additive EPSPs by slightly enhancing the probabilistic model.

In our experiments with static input patterns we typically use the following basis scheme to encode the external input variables 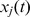 by populations of stochastic spiking neurons 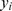: at every point in time 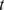 there is exactly one neuron 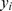 in every group 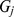 that represents the instantaneous value of 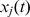. We call this neuron the active neuron of the group, whereas all other neurons of the group are inactive. During the time where a neuron 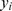 is active it fires stochastically according to a Poisson processes with a certain constant or oscillating rate. The inactive neurons, however, remain silent, i.e. they fire with a rate near 0. Although not explicitly modeled here, such an effect can result from strong lateral inhibition in the input populations. This scheme certainly fulfills the definition in Eq. (8).

Here and in the following we will write 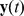 to denote the input activation through the EPSPs of the network model, and 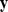 to denote a variable in the probabilistic model, which models the distribution of 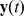 over all time points 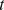. We will also use notations like 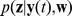, which refers to the variable 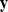 in the probabilistic model taking on the value 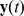. We can then reformulate the abstract probabilistic model 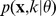 using the above population codes that define the binary variable vectors 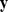 and 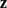, with 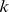 s.t. 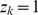 as:
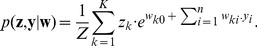
(9)Under the normalization conditions
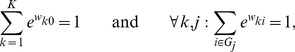
(10)the normalization constant 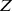 vanishes and the parametrization of the distribution simplifies to 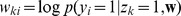 and 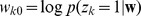. Even for non-normalized weights, the definition in Eq. (9) still represents the same type of distribution, although there is no more one-to-one mapping between the weights 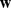 and the parameters of the graphical model (see Methods for details). Note also that such log-probabilities are exactly (up to additive constants) the local equilibrium points in Eq. (6) of the STDP rule in Fig. 2. In the section “STDP approximates Expectation Maximization” we will discuss in detail how this leads to unsupervised learning of a generative model of the input data in a WTA circuit.

#### Spike-based Bayesian computation

We can now formulate an exact link between the above generative probabilistic model and our neural network model of a simplified spike-based WTA circuit. We show that at any point in time 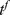 at which the network generates an output spike, the relative firing probabilities 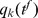 of the output neurons 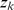 as in Eq. (4), are equal to the posterior distribution of the hidden cause 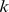, given the current evidences encoded in the input activations 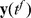. For a given input 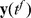 we use Bayes' rule to calculate the posterior probability of cause 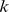 as 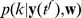. We can identify the prior 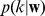 with the excitabilities 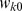 of the neurons. The log-likelihood 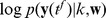 of the current evidences given the cause 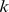 corresponds to the sum of excitatory EPSPs, which depend on the synaptic weights 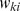. This leads to the calculation
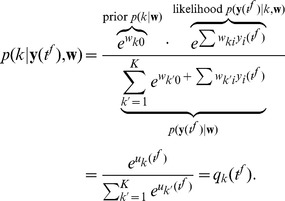
(11)This shows that at all times 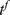 every spike from the WTA circuit represents one sample of the instantaneous posterior distribution 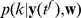.

The crucial observation, however, is that this relation is valid at any point in time, independently of the inhibitory signal 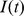. It is only the ratio between the quantities 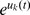 that determines the relative firing probabilities 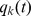 of the neurons 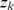.

#### Background oscillations and learning with missing values

We will now show that for the case of a low average input firing rate, a modulation of the firing rate can be beneficial, as it can synchronize firing of pre- and post-synaptic neurons. Each active neuron then fires according to an inhomogeneous Poisson process, and we assume for simplicity that the time course of the spike rate for all neurons follows the same oscillatory (sinusoidal) pattern around a common average firing rate. Nevertheless the spikes for each 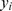 are drawn as samples from independent processes. In addition, let the common inhibition signal 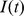 be modulated by an additional oscillatory current 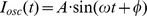 with amplitude 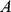, oscillation frequency 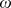 (same as for the input oscillation), and phase shift 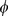. Due to the increased number of input neurons firing simultaneously, and the additional background current, pre- and post-synaptic firing of active neurons will synchronize. The frequency of the background oscillation can be chosen in principle arbitrarily, as long as the number of periods per input example is constant. Otherwise the network will weight different input examples by the number of peaks during presentation, which might lead to learning of a different generative model.

The effect of a synchronization of pre- and post-synaptic firing can be very beneficial, since at low input firing rates it might happen that none of the input neurons in a population of neurons encoding an external variable 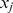 fires within the integration time window of length 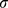 of output neurons 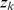. This corresponds to learning with missing attribute values for 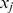, which is known to impair learning performance in graphical models [48]. Our novel interpretation is therefore that background oscillations can reduce the percentage of missing values by synchronizing presynaptic firing rates. This agrees with previous studies, which have shown that it is easier for single detector neurons learning with phenomenological STDP rules to detect spike patterns embedded in a high-dimensional input stream, if the patterns are encoded relative to a background oscillation [49], or the patterns consist of dense and narrow bursts of synchronous activity [50]. These results still hold if only a small part of the afferents participates in the pattern, or spikes from the pattern are missing, since the increased synchrony facilitates the identification of the pattern. Although we show in experiments that this increased synchronization can improve the learning performance of spike-based probabilistic learners in practice, it is important to note that background oscillations are not necessary for the theory of spike-based Expectation Maximization to hold. Also, brain oscillations have previously been associated with various fundamental cognitive functions like e.g. attention, memory, consciousness, or neural binding. In contrast, our suggested role for oscillations as a mechanism for improving learning and inference with missing values is very specific within our framework, and although some aspects are compatible with higher-level theories, we do not attempt here to provide alternative explanations for these phenomena.

Our particular model of oscillatory input firing rates leaves the average firing rates unchanged, hence the effect of oscillations does not simply arise due to a larger number of input or output spikes. It is the increased synchrony of input and output spikes by which background oscillations can facilitate learning for tasks in which inputs have little redundancy, and missing values during learning thus would have a strong impact. We demonstrate this in the following experiment, where a common background oscillation for the input neurons 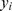 and the output neurons 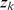 significantly speeds up and improves the learning performance. In other naturally occurring input distributions with more structured inputs, oscillations might not improve the performance.

### Example 1: Learning of probabilistic models with STDP

**Figure 3:**
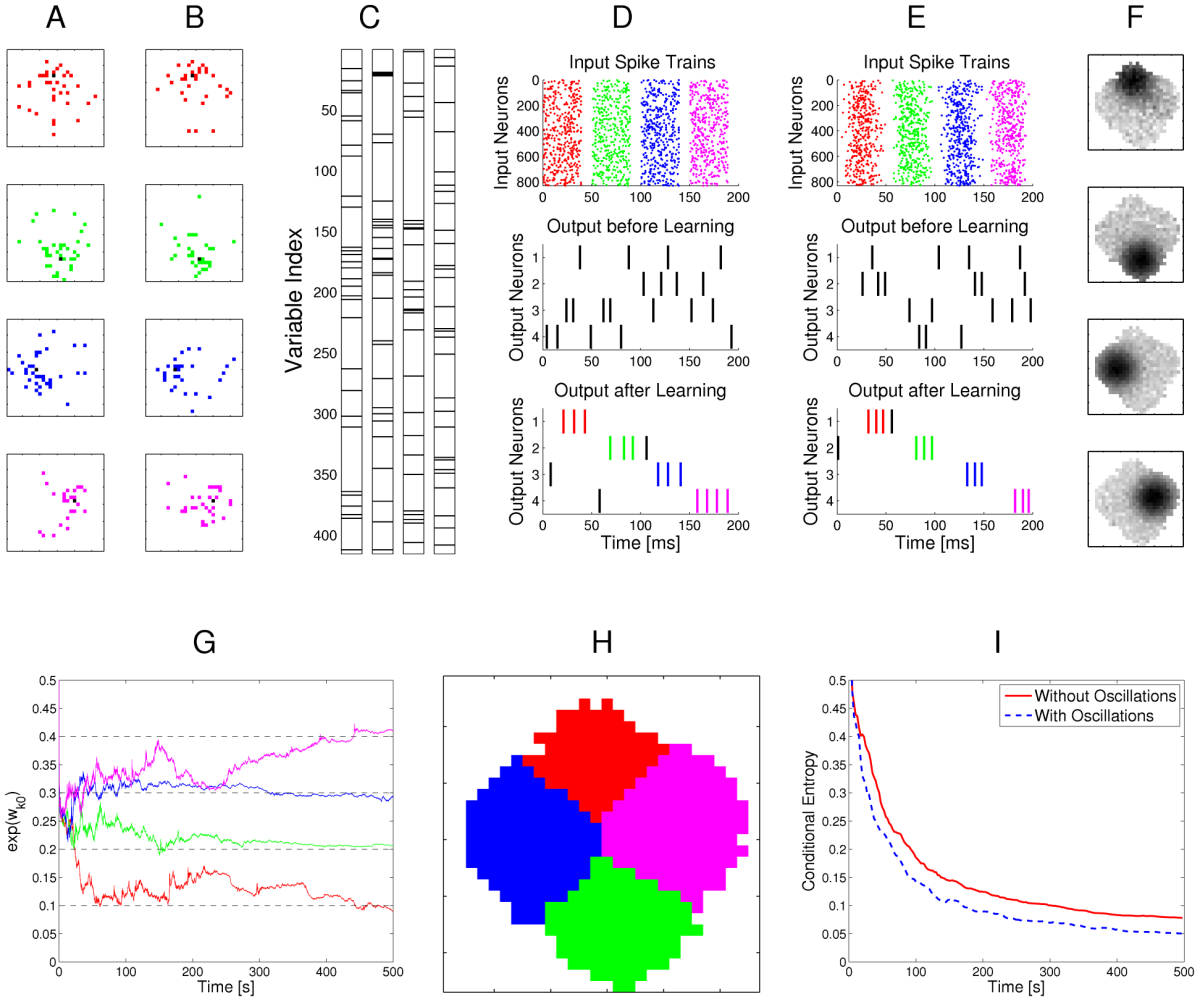
Example for the emergence of Bayesian computation through STDP and adaptation of neural excitability. **A, B**: Visualization of hidden structure in the spike inputs 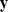 shown in D, E: Each row in panels A and B shows two results of drawing pixels from the same Gauss distribution over a 28×28 pixel array. Four different Gauss distributions were used in the four rows, and the location of their center represents the latent variable behind the structure of the input spike train. **C**: Transformation of the four 2D images in B into four linear arrays, resulting from random projections from 2D locations to 1D indices. Black lines indicate active pixels, and pixels that were active in less than 4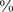 of all images were removed before the transformation (these pixels are white in panel H). By the random projection, both the 2D structure of the underlying pixel array and the value of the latent variable are hidden when the binary 1D vector is encoded through population coding into the spike trains 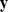 that the neural circuit receives. **D**: Top row: Spike trains from 832 input neurons that result from the four linear patterns shown in panel C (color of spikes indicates which of the four hidden processes had generated the underlying 2D pattern, after 50 ms another 2D pattern is encoded). The middle and bottom row show the spike output of the four output neurons at the beginning and after 500 s of unsupervised learning with continuous spike inputs (every 50 ms another 2D pattern was randomly drawn from one of the 4 different Gauss distributions, with different prior probabilities of 0.1, 0.2, 0.3, and 0.4.). Color of spikes indicates the emergent specialization of the four output neurons on the four hidden processes for input generation. Black spikes indicate incorrect guesses of hidden cause. **E**: Same as D, but with a superimposed 20 Hz oscillation on the firing rates of input neurons and membrane potentials of the output neurons. Fewer error spikes occur in the output, and output spikes are more precisely timed. **F**: Internal models (weight vectors 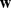) of output neurons 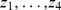 after learning (pixel array). **G**: Autonomous learning of priors 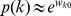, that takes place simultaneously with the learning of internal models. **H**: Average “winner” among the four output neurons for a test example (generated with equal probability by any of the 4 Gaussians) when a particular pixel was drawn in this test example, indicating the impact of the learned priors on the output response. **I**: Emergent discrimination capability of the output neurons during learning (red curve). The dashed blue curve shows that a background oscillation as in E speeds up discrimination learning. Curves in G and I represent averages over 20 repetitions of the learning experiment.

Fig. 3 demonstrates the emergence of Bayesian computation in the generic network motif of Fig. 1A in a simple example. Spike inputs 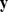 (top row of Fig. 3D) are generated through four different hidden processes (associated with four different colors). Each of them is defined by a Gauss distribution over a 2D pixel array with a different center, which defines the probability of every pixel to be on. Spike trains encode the current value of a pixel by a firing rate of 25 Hz or 0 Hz for 40 ms. Each pixel was encoded by two input neurons 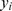 via population coding, exactly one of them had a firing rate of 25 Hz for each input image. A 10 ms period without firing separates two images in order to avoid overlap of EPSPs for input spikes belonging to different input images.

After unsupervised learning with STDP for 500 s (applied to continuous streams of spikes as in panel D of Fig. 3) the weight vectors shown in Fig. 3F (projected back into the virtual 2D input space) emerged for the four output neurons 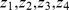, demonstrating that these neurons had acquired internal models for the four different processes that were used to generate inputs. The four different processes for generating the underlying 2D input patterns had been used with different prior probabilities (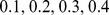). Fig. 3G shows that this imbalance resulted in four different priors 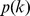 encoded in the biases 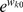 of the neurons 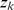. When one compares the unequal sizes of the colored areas in Fig. 3H with the completely symmetric internal models (or likelihoods) of the four neurons shown in panel F, one sees that their firing probability approximates a posterior over hidden causes that results from multiplying their learned likelihoods with their learned priors. As a result, the spike output becomes sparser, and almost all neurons only fire when the current input spikes are generated by that one of the four hidden processes on which they have specialized (Fig. 3D, bottom row). In Fig. 3I the performance of the network is quantified over time by the normalized conditional entropy 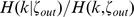, where 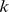 is the correct hidden cause of each input image 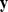 in the training set, and 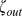 denotes the discrete random variable defined by the firing probabilities of output neurons 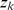 for each image under the currently learned model. Low conditional entropy indicates that each neuron learns to fire predominantly for inputs from one class. Fig. 3E as well as the dashed blue line in Fig. 3I show that the learning process is improved when a common background oscillation at 20 Hz is superimposed on the firing rate of input neurons and the membrane potential of the output neurons, while keeping the average input and output firing rates constant. The reason is that in general it may occur that an output neuron 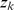 receives during its integration time window (40 ms in this example) no information about the value of a pixel (because neither the neuron 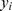 that has a high firing rate for 40 ms if this pixel is black, nor the associated neuron 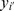 that has a high firing rate if this pixel is white fire during this time window). A background oscillation reduces the percentage of such missing values by driving presynaptic firing times together (see top row of Fig. 3E). Note that through these oscillations the overall output firing rate 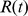 fluctuates strongly, but since the same oscillation is used consistently for all four types of patterns, the circuit still learns the correct distribution of inputs.

This task had been chosen to become very fast unsolvable if many pixel values are missing. Many naturally occurring input distributions, like the ones addressed in the subsequent computer experiments, tend to have more redundancy, and background oscillations did not improve the learning performance for those.

### STDP approximates Expectation Maximization

In this section we will develop the link between the unsupervised learning of the generative probabilistic model in Fig. 1B and the learning effect of STDP as defined in our spiking network model in Fig. 1A. Starting from a learning framework derived from the concept of Expectation Maximization [31], we show that the biologically plausible STDP rule from Fig. 2 can naturally approximate a stochastic, online version of this optimization algorithm. We call this principle SEM (spike-based EM).

SEM can be viewed as a bootstrapping procedure. The relation between the firing probabilities of the neurons within the WTA circuit and the continuous updates of the synaptic weights with our STDP rule in Eq. (5) drive the initially random firing of the circuit in response to an input 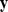 towards learning the correct generative model of the input distribution. Whenever a neuron 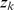 fires in response to 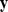, the STDP rule increases the weights 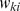 of synapses from those presynaptic neurons 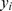 that had fired shortly before 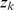. In absence of a recent presynaptic spike from 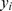 the weight 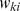 is decreased. As a consequence, when next a pattern similar to 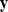 is presented, the probability for the same 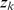 to fire and further adapt its weights, is increased. Since 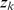 becomes more of an “expert” for one subclass of input patterns, it actually becomes less likely to fire for non-matching patterns. The competition in the WTA circuit ensures that other 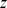-neurons learn to specialize for these different input categories.

In the framework of Expectation Maximization, the generation of a spike in a 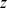-neuron creates a sample from the currently encoded posterior distribution of hidden variables, and can therefore be viewed as the stochastic Expectation, or 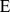-step. The subsequent application of STDP to the synapses of this neuron can be understood as an approximation of the Maximization, or 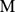-step. The online learning behavior of the network can be understood as a stochastic online EM algorithm.

#### Learning the parameters of the probability model by EM

The goal of learning the parametrized generative probabilistic model 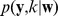 is to find parameter values 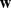, such that the marginal distribution 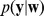 of the model distribution approximates the actual stationary distribution of spike inputs 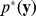 as closely as possible. We define 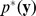 as the probability to observe the activation vector 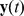 at some point 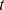 in time (see Eq. (72) in Methods for a precise mathematical definition). The learning task can thus be formalized as the minimization of the Kullback-Leibler divergence between the two distributions, 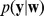 and 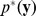. A mathematically equivalent formulation is the maximization of the expected likelihood 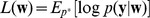 of the inputs 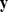, drawn from 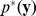. The parametrization of the generative probabilistic model 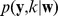 is highly redundant, i.e. for every 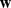 there is a continuous manifold of 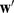, that all define identical generative distributions 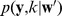 in Eq. (24). There is, however, exactly one 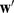 in this sub-manifold of the weight space that fulfills the normalization conditions in Eq. (10). By imposing the normalization conditions as constraints to the maximization problem, we can thus find unique local maxima (see “Details to Learning the parameters of the probability model by EM” in Methods).

The most common way to solve such unsupervised learning problems with hidden variables is the mathematical framework of Expectation Maximization (EM). In its standard form, the EM algorithm is a batch learning mechanism, in which a fixed, finite set of 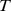 instances of input vectors 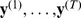 is given, and the task is to find the parameter vector 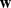 that maximizes the log-likelihood 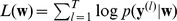 of these 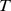 instances to be generated as independent samples by the model 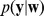.

Starting from a random initialization for 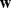, the algorithm iterates between E-steps and M-steps. In the E-steps, the current parameter vector 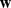 is used to find the posterior distributions of the latent variables 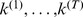, each given by 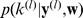.

In the M-steps a new parameter vector 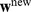 is computed, which maximizes the expected value of the complete-data log-likelihood function, subject to the normalization constraints in Eq. (10). The analytical solution for this M-step (compare [32]) is given by
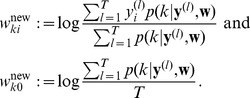
(12)The iterated application of this update procedure is guaranteed to converge to a (local) maximum of 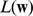 [31]. It is obvious that 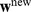 fulfills the desired normalization conditions in Eq. (10) after every update.

Although the above deterministic algorithm requires that the same set of 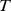 training examples is re-used for every EM iteration, similar results also hold valid for online learning scenarios. In an online setup new samples 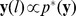 are drawn from the input distribution at every iteration, which is closer to realistic neural network learning settings. Instead of analytically computing the expected value of the complete-data log-likelihood function, a Monte-Carlo estimate is computed using the samples 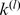, drawn according to their posterior distribution 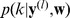. Even though additional stochastic fluctuations are introduced due to the stochastic sampling process, this stochastic EM algorithm will also converge to a stable result in the limit of infinite iterations, if the number of samples 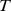 is increased with every iteration [51].

In order to simplify the further notation we introduce the augmented input distribution 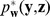 from which we can sample pairs 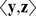 and define
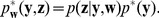
(13)


Sampling pairs 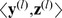 with 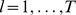 from 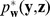 corresponds to online sampling of inputs, combined with a stochastic E-step. The subsequent M-step
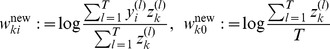
(14)essentially computes averages over all 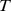 samples: 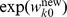 is the average of the variable 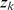; 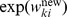 is a conditional average of 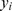 taken over those instances in which 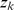 is 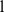.

The expected value of the new weight vector after one iteration, i.e., the sampling E-step and the averaging M-step, can be expressed in a very compact form based on the augmented input distribution as
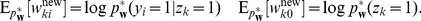
(15)


A necessary condition for a point convergence of the iterative algorithm is a stable equilibrium point, i.e. a value 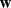 at which the expectation of the next update 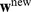 is identical to 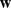. Thus we arrive at the following necessary implicit condition for potential convergence points of this stochastic algorithm.
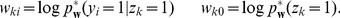
(16)This very intuitive implicit “solution” is the motivation for relating the function of the simple STDP learning rule (solid red line in Fig. 2) in the neural circuit shown in Fig. 1A to the framework of EM.

#### Spike-based Expectation Maximization

In order to establish a mathematically rigorous link between the STDP rule in Fig. 2 in the spike-based WTA circuit and stochastic online EM we identify the functionality of both the E- and the M-steps with the learning behavior of the spiking WTA-circuit with STDP.

In a biologically plausible neural network setup, one cannot assume that observations are stored and computations necessary for learning are deferred until a suitable sample size has been reached. Instead, we relate STDP learning to online learning algorithms in the spirit of Robbins-Monro stochastic approximations, in which updates are performed after every observed input.

At an arbitrary point in time 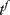 at which any one neuron 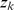 of the WTA circuit fires, the posterior 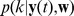 according to Eq. (4) gives the probability that the spike at this time 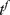 has originated from the neuron with index 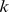. The pair 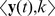 can therefore be seen as a sample from the augmented input distribution 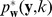. Hence, we can conclude that the generation of a spike by the WTA circuit corresponds to the generation of samples 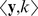 during the E-step. There are additional conditions on the inhibition signal 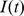 that have to be met in order to generate unbiased samples 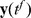 from the input distribution 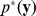. These are discussed in depth in the section “Role of the Inhibition”, but for now let us assume that these conditions are fulfilled.

The generation of a spike in the postsynaptic neuron 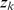 triggers an STDP update according to Eq. (5) in all synapses from incoming presynaptic neurons 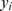, represented by weights 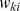. We next show that the biologically plausible STDP rule in Eq. (5) (see also Fig. 2) together with the rule in Eq. (7) can be derived as approximating the M-step in stochastic online EM.

The update in Eq. (14) suggests that every synapse 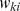 collects the activation statistics of its input 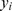 (the presynaptic neuron), given that its output 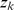 (the postsynaptic neuron) fires. These statistics can be gathered online from samples of the augmented input distribution 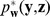.

From this statistical perspective each weight can be interpreted as 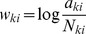, where 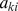 and 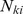 are two local virtual counters in each synapse. 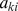 represents the number of the events 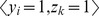 and 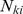 represents the number of the events 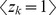, i.e. the postsynaptic spikes. Even though all virtual counters 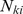 within one neuron 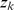 count the same postsynaptic spikes, it is easier to think of one individual such counter for every synapse. If we interpret the factor 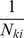 as a local learning rate 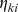, we can derive Eq. (5) (see Methods) as the spike-event triggered stochastic online learning rule 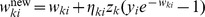 that approximates in the synapse 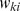 the log of the running average of 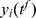 at the spiking times of neuron 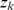. The update formula shows that 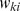 is only changed, if the postsynaptic neuron 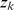 fires, whereas spike events of other neurons 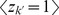 with 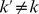 are irrelevant for the statistics of 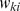. Thus the learning rule is purely local for every synapse 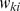; it only has to observe its own pre- and postsynaptic signals. Additionally we show in the Methods section “Adaptive learning rates with Variance tracking” a very efficient heuristic how the learning rate 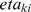 can be estimated locally.

Analogously we can derive the working mechanism of the update rule in Eq. (7) as updates of the log of a fraction at the respective points in time.

The simple STDP rules in Eq. (5) and Eq. (7) thus approximate the M-step in a formal generative probabilistic model with local, biologically plausible computations. It remains to be shown that these STDP rules actually drive the weights 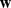 to converge to the target points in Eq. (16) of the stochastic EM algorithm.

We can conclude from the equilibrium conditions of the STDP rule in Eq. (6) that convergence can only occur at the desired local maxima of the likelihood 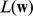 subject to the normalization constraints. However, it remains to be shown that the update algorithm converges at all and that there are no limit cycles.

#### Proof of convergence

Even though we successfully identified the learning behavior of the simple STDP rule (Fig. 2) in the circuit model with the E- and the M-steps of the EM algorithm, this is not yet sufficient for a complete proof of convergence for the whole learning system. Not only are the single updates just approximations to the M-step, these approximations, in addition, violate the normalization conditions in Eq. (10). Although the system - as we will show - converges towards normalized solutions, there is always a stochastic fluctuation around the normalization conditions. One can therefore not simply argue that Eq. (5) implements a stochastic version of the generalized EM algorithm; instead, we have to resort to the theory of stochastic approximation algorithms as presented in [52]. Under some technical assumptions (see Methods) we can state

Theorem 1: *The algorithm in* *Eq. (5*,*7)* *updates* 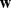 *in a way that it converges with probability 1 to the set of local maxima of the likelihood function* 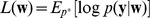 *, subject to the normalization constraints in* *Eq. (10)* *.*

The detailed proof, which is presented in Methods, shows that the expected trajectory of the weight vector 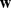 is determined by two driving forces. The first one is a normalization force which drives 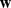 from every arbitrary point towards the regime where 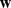 is normalized. The second force is the real learning force that drives 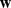 to a desired maximum of 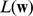. However, this interpretation of the learning force is valid only if 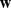 is sufficiently close to normalized.

### The role of the inhibition

We have previously shown that the output spikes of the WTA circuit represent samples from the posterior distribution in Eq. (11), which only depends on the ratios between the membrane potentials 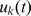. The rate at which these samples are produced is the overall firing rate 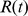 of the WTA circuit and can be controlled by modifying the common inhibition 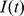 of the neurons 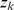.

Although any time-varying output firing rate 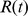 produces correct samples from the posterior distribution in Eq. (11) of 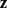, for learning we also require that the input patterns 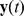 observed at the spike times are unbiased samples from the true input distribution 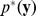. If this is violated, some patterns coincide with a higher 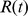, and thus have a stronger influence on the learned synaptic weights. In Methods we formally show that 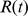 acts as a multiplicative weighting of the current input 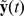, and so the generative model will learn a slightly distorted input distribution.

An unbiased set of samples can be obtained if 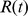 is independent of the current input activation 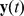, e.g. if 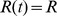 is constant. This could in theory be achieved if we let 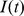 depend on the current values of the membrane potentials 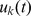, and set 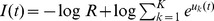. Such an immediate inhibition is commonly assumed in rate-based soft-WTA models, but it seems implausible to compute this in a spiking neuronal network, where only spikes can be observed, but not the presynaptic membrane potentials.

However, our results show that a perfectly constant firing rate is not a prerequisite for convergence to the right probabilistic model. Indeed we can show that it is sufficient that 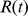 and 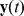 are stochastically independent, i.e. 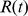 is not correlated to the appearance of any specific value of 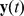. Still this might be difficult to achieve since the firing rate 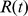 is functionally linked to the input 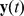 by 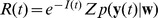, but it clarifies the role of the inhibition 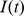 as de-correlating 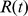 from the input 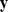, at least in the long run.

One possible biologically plausible mechanism for such a decorrelation of 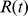 and 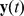 is an inhibitory feedback from a population of neurons that is itself excited by the neurons 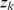. Such WTA competition through lateral inhibition has been studied extensively in the literature [16], [33]. In the implementation used for the experiments in this paper every spike from the 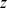-neurons causes an immediate very strong inhibition signal that lasts longer than the refractory period of the spiking neuron. This strong inhibition decays exponentially and is overlaid by a noise signal with high variability that follows an Ornstein-Uhlenbeck process (see “Inhibition Model in Computer Simulations” in Methods). This will render the time of the next spike of the system almost independent of the value of 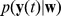.

It should also be mentioned that a slight correlation between 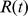 and 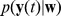 may be desirable, and 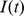 might also be externally modulated (for example through attention, or neuromodulators such as Acetylcholin), as an instrument of selective input learning. This might lead e.g. to slightly higher firing rates for well-known inputs (high 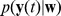), or salient inputs, as opposed to reduced rates for unknown arbitrary inputs. In general, however, combining online learning with a sampling rate 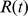 that is correlated to 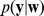 may lead to strange artifacts and might even prohibit the convergence of the system due to positive feedback effects. A thorough analysis of such effects and of possible learning mechanisms that cope with positive feedback effects is the topic of future research.

Our theoretical analysis sheds new light on the requirements for inhibition in spiking WTA-like circuits to support learning and Bayesian computation. Inhibition does not only cause competition between the excitatory neurons, but also regulates the overall firing rate 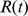 of the WTA circuit. Variability in 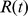 does not influence the performance of the circuit, as long as there is no systematic dependence between the input and 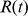.

### Continuous-time interpretation with realistically shaped EPSPs

In our previous analysis we have assumed a simplified non-additive step-function model for the EPSP. This allowed us to describe all input evidence within the last time window of length 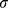 by one binary vector 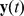, but required us to assume that no two neurons within the same group 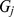 fired within that period. We will now give an intuitive explanation to show that this restriction can be dropped and present an interpretation for additive biologically plausibly shaped EPSPs as inference in a generative model.

The postsynaptic activation 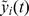 under an additive EPSPs is given by the convolution
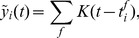
(17)where 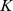 describes an arbitrarily shaped kernel, e.g. an 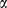-shaped EPSP function which is the difference of two exponential functions (see [37]) with different time constants. We use 1 ms for the rise and 15 ms for the decay in our simulations. 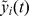 replaces 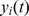 in Eq. (2) in the computation of the membrane potential 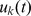 of our model neurons. We can still understand the firing of neurons in the WTA circuit according to the relative firing probabilities 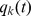 in Eq. (4) as Bayesian inference. To see this, we imagine an extension of the generative probabilistic model 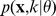 in Fig. 1B, which contains multiple instances of 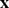, exactly one for every input spike from all input neurons 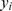. For a fixed common hidden cause 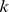, all instances of 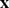 are conditionally independent of each other, and have the same conditional distributions for each 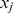 (see Methods for the full derivation of the extended probabilistic model). According to the definition in Eq. (8) of the population code every input spike represents evidence that 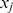 in an instance 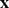 should take on a certain value. Since every spike contributes only to one instance, any finite input spike pattern can be interpreted as valid evidence for multiple instances of inputs 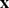.

The inference of a single hidden cause 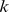 in such extended graphical model from multiple instances of evidence is relatively straightforward: due to the conditional independence of different instances, we can compute the input likelihood for any hidden cause simply as the product of likelihoods for every single evidence. Inference thus reduces to counting how often every possible evidence occurred in all instances 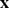, which means counting the number of spikes of every 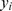. Since single likelihoods are implicitly encoded in the synaptic weights 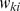 by the relationship 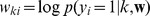, we can thus compute the complete input likelihood by adding up step-function like EPSPs with amplitudes corresponding to 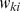. This yields correct results, even if one input neuron spikes multiple times.

In the above model, the timing of spikes does not play a role. If we want to assign more weight to recent evidence, we can define a heuristic modification of the extended graphical model, in which contributions from spikes to the complete input log-likelihood are linearly interpolated in time, and multiple pieces of evidence simply accumulate. This is exactly what is computed in 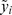 in Eq. (17), where the shape of the kernel 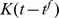 defines how the contribution of an input spike at time 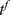 evolves over time. Defining 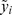 as the weight for the evidence of the assignment of 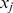 to value 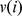, it is easy to see (and shown in detail in Methods) that the instantaneous output distribution 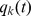 represents the result of inference over causes 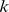, given the time-weighted evidences of all previous input spikes, where the weighting is done by the EPSP-function 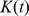. Note that this evidence weighting mechanism is not equivalent to the much more complex mechanism for inference in presence of uncertain evidence, which would require more elaborate architectures than our feed-forward WTA-circuit. In our case, past evidence does not become uncertain, but just less important for the inference of the instantaneous hidden cause 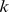.

We can analogously generalize the spike-triggered learning rule in Eq. (5) for continuous-valued input activations 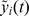 according to Eq. (17):
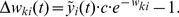
(18)The update of every weight 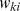 is triggered when neuron 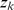, i.e. the postsynaptic neuron, fires a spike. The shape of the LTP part of the STDP curve is determined by the shape of the EPSP, defined by the kernel function 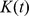. The positive part of the update in Eq. (18) is weighted by the value of 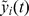 at the time of firing the postsynaptic spike. Negative updates are performed if 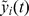 is close to zero, which indicates that no presynaptic spikes were observed recently. The complex version of the STDP curve (blue dashed curve in Fig. 1B), which resembles more closely to the experimentally found STDP curves, results from the use of biologically plausible 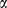-shaped EPSPs. In this case, the LTP window of the weight update decays with time, following the shape of the 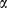-function. This form of synaptic plasticity was used in all our experiments. If EPSPs accumulate due to high input stimulation frequencies, the resulting shape of the STDP curve becomes even more similar to previously observed experimental data, which is investigated in detail in the following section.

The question remains, how this extension of the model and the heuristics for time-dependent weighting of spike contributions affect the previously derived theoretical properties. Although the convergence proof does not hold anymore under such general conditions we can expect (and show in our Experiments) that the network will still show the principal behavior of EM under fairly general assumptions on the input: we have to assume that the instantaneous spike rate of every input group 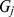 is not dependent on the value of 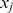 that it currently encodes, which means that the total input spike rate must not depend on the hidden cause 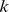. Note that this assumption on every input group is identical to the desired output behavior of the WTA circuit according to the conditions on the inhibition as derived earlier. This opens up the possibility of building networks of recursively or hierarchically connected WTA circuits. Note also that the grouping of inputs into different 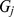 is only a notational convenience. The neurons in the WTA circuit do not have to know which inputs are from the same group, neither for inference nor for learning, and can thus treat all input neurons equally.

### Relationship to experimental data on synaptic plasticity

In biological STDP experiments that induce pairs of pre- and post-synaptic spikes at different time delays, it has been observed that the shape of the plasticity curve changes as a function of the repetition frequency for those spike pairs [40]. The observed effect is that at very low frequencies no change or only LTD occurs, a “classical” STDP window with timing-dependent LTD and LTP is observed at intermediate frequencies around 20 Hz, and at high frequencies of 40 Hz or above only LTP is observed, independently of which spikes comes first.

Although our theoretical model does not explicitly include a stimulation-frequency dependent term like other STDP models (e.g. [53]), we can study empirically the effect of a modification of the frequency of spike-pairing. We simulate this for a single synapse, at which we force pre- and post-synaptic spikes with varying time differences 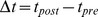, and at fixed stimulation frequencies 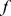 of either 1 Hz, 20 Hz, or 40 Hz. Modeling EPSPs as 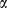-kernels with time constants of 1 ms for the rise and 15 ms for the decay, we obtain the low-pass filtered signals 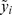 as in Eq. (17), which grow as EPSPs start to overlap at higher stimulation frequencies. At the time of a post-synaptic spike we compute the synaptic update according to the rule in Eq. (18), but keep both the weight and the learning rate fixed (at 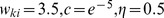) to distinguish timing-dependent from weight-dependent effects.

**Figure 4:**
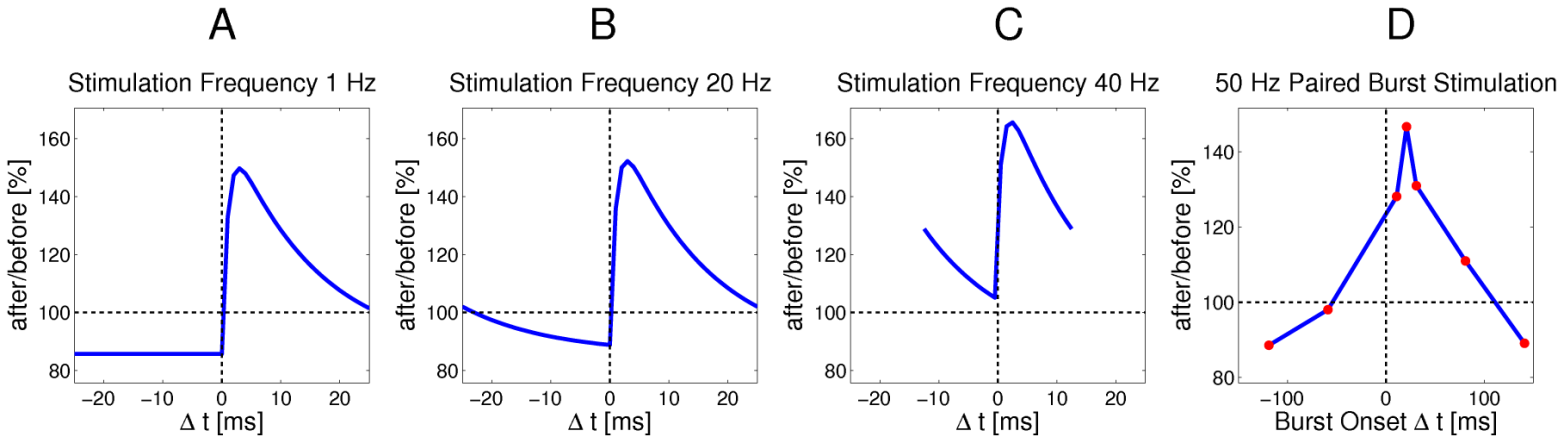
Relationship between the continuous-time SEM model and experimental data on synaptic plasticity. **A–C**: The effect of the continuous-time plasticity rule in Eq. (18) at a single synapse for different stimulation frequencies and different time-differences between pre- and post-synaptic spike pairs. Only time-intervals without overlapping pairs are shown. **A**: For very low stimulation frequencies (1 Hz) the standard shape of the complex learning rule from Fig. 2 is recovered. **B**: At a stimulation frequency of 20 Hz the plasticity curve shifts more towards LTP, and depression is no longer time independent, due to overlapping EPSPs. **C**: At high stimulation frequencies of 40 Hz or above, the STDP curve shifts towards only LTP, and thus becomes similar to a rate-based Hebbian learning rule. **D**: Cumulative effect of pre- and post-synaptic burst stimulation (50 Hz bursts of 5 pre-synaptic and 4 post-synaptic spikes) with different onset delays of -120, -60, 10, 20, 30, 80 and 140 ms (time difference between the onsets of the post- and pre-synaptic bursts). As in [55], the amount of overlap between bursts determines the magnitude of LTP, rather than the exact temporal order of spikes.

In Fig. 4A we observe that, as expected, at low stimulation frequencies (1 Hz) the standard shape of the complex STDP rule in Eq. (18) from Fig. 2 is recovered, since there is no influence from previous spikes. The shift towards pure LTD that is observed in biology [40] would require an additional term that depends on postsynaptic firing rates like in [53], and is a topic of future research. However, note that in biology this shift to LTD was observed only in paired recordings, neglecting the cooperative effect of other synapses, and other studies have also reported LTP at low stimulation frequencies [43]. At higher stimulation frequencies (20 Hz in Fig. 4B) the EPSPs from different pre-synaptic spikes start to overlap, which results in larger 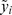 compared with isolated pre-synaptic spikes. We also see that the LTD part of the STDP window becomes timing-dependent (due to overlapping EPSPs), and thus the shape of the STDP curve becomes similar to standard models of STDP and observed biological data [43], [54]. For even higher stimulation frequencies the STDP window shifts more and more towards LTP (see Fig. 4B and C). This is in good accordance with observations in biology [40]. Also in agreement with biological data, the minimum of the update occurs around 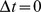, because there the new 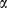-kernel EPSP is not yet effective, and the activation due to previous spikes has decayed maximally.

Another effect that is observed in hippocampal synapses when two neurons are stimulated with bursts, is that the magnitude of LTP is determined mostly by the amount of overlap between the pre- and post-synaptic bursts, rather than the exact timing of spikes [55]. In Fig. 4D we simulated this protocol with our continuous-time SEM rule for different onset time-differences of the bursts, and accumulated the synaptic weight updates in response to 50 Hz bursts of 5 pre-synaptic and 4 post-synaptic spikes. We performed this experiment for the same onset time differences used in Fig. 3 of [55], and found qualitatively similar results. For long time-differences, when EPSPs have mostly decayed, we observed an LTD effect, which was not observed in biology, but can be attributed to differences in synaptic time constants between biology and simulation.

These results suggest that our STDP rule derived from theoretical principles exhibits several of the key properties of synaptic plasticity observed in nature, depending on the encoding of inputs. This is quite remarkable, since these properties are not explicitly part of our learning rule, but rather emerge from a simpler rule with strong theoretical guarantees. Other phenomenological [56], [57] or mechanistic models of STDP [58] also show some of these characteristics, but come without such theoretical properties. The functional consequence of reproducing such key biological characteristics of STDP is that our new learning rule also exhibits most of the key functional properties of STDP, like e.g. strengthening synapses of inputs that are causally involved in firing the postsynaptic neuron, while pruning the connections that do not causally contribute to postsynaptic firing [10], [13]. At low and intermediate firing rates our rule also shifts the onset of postsynaptic firing towards the start of repeated spike patterns [49], [50], [59], while depressing synapses that only become active for a pattern following the one for which the post-synaptic neuron is responsive. If patterns change quickly, then the stronger depression for presynaptic spikes with small 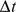 in Fig. 4B enhances the capability of the WTA to discriminate such patterns. With simultaneous high frequency stimulation (Fig. 4C and D) we observe that only LTP occurs, which is due to the decay of EPSPs not being fast enough to allow depression. In this scenario, the learning rule is less sensitive to timing, and rather becomes a classical Hebbian measure of correlations between pre- and post-synaptic firing rates. However, since inputs are encoded in a population code we can assume that the same neuron is not continuously active throughout, and so even at high firing rates for active input neurons, the synapses that are inactive during postsynaptic firing will still be depressed, which means that convergence to an equilibrium value is still possible for all synapses.

It is a topic of future research which effects observed in biology can be reproduced with more complex variations of the spike-based EM rule that are also dependent on postsynaptic firing rates, or whether existing phenomenological models of STDP can be interpreted in the probabilistic EM framework. In fact, initial experiments have shown that several variations of the spike-based EM rule can lead to qualitatively similar empirical results for the learned models in tasks where the input spike trains are Poisson at average or high rates over an extended time window (such as in Fig. 3). These variations include weight-dependent STDP rules that are inversed in time, symmetrical in time, or have both spike timing-dependent LTD and LTP. Such rules can converge towards the same equilibrium values as the typical causal STDP rule. However, they will behave differently if inputs are encoded through spatio-temporal spike patterns (as in Example 4: Detection of Spatio-Temporal Spike Patterns). Further variations can include short-term plasticity effects for pre-synaptic spikes, as observed and modeled in [60], which induce a stimulation-frequency dependent reduction of the learning rate, and could thus serve as a stabilization mechanism.

### Spike-timing dependent LTD

Current models of STDP typically assume a “double-exponential” decaying shape of the STDP curve, which was first used in [54] to fit experimental data. This is functionally different from the shape of the complex STDP curve in Fig. 2 and Eq. (5), where the LTD part is realized by a constant timing-independent offset.

Although not explicitly covered by the previously presented theory of SEM, the same analytical tools can be used to explain functional consequences of timing-dependent LTD in our framework. Analogous to our approach for the standard SEM learning rule, we develop (in Methods) an extension of the simple step-function STDP rule from Fig. 2 with timing-dependent LTD, which is easier to analyze. We then generalize these results towards arbitrarily shaped STDP curves. The crucial result is that as long as the spike-timing dependent LTD rule retains the characteristic inversely-exponential weight-dependent relationship between the strengths of LTP and LTD that was introduced for standard SEM in Eq. (5), an equilibrium property similar to Eq. (6) still holds (see Methods for details). Precisely speaking, the new equilibrium will be at the difference between the logarithms of the average presynaptic spiking probabilities *before* and *after* the postsynaptic spike. This shows that spike-timing dependent LTD also yields synaptic weights that can be interpreted in terms of log-probabilities, which can thus be used for inference.

The new rule emphasizes contrasts between the current input pattern and the immediately following activity. Still, the results of the new learning rule and the original rule from Eq. (5) in our experiments are qualitatively similar. This can be explained from a stochastic learning perspective: at any point in time the relative spiking probabilities of excitatory neurons in the WTA circuit in Eq. (4) depend causally on the weighted sums of preceding presynaptic activities 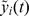. However, they clearly do not depend on future presynaptic activity. Thus, the postsynaptic neuron will learn through SEM to fire for increasingly similar stochastic realizations of presynaptic input 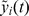, whereas the presynaptic activity pattern following a postsynaptic spike will become more variable. In the extreme case where patterns are short and separated by noise, there will be no big difference between input patterns following firing of any of the WTA neurons, and so their relevance for the competition will become negligible.

Experimental evidence shows that the time constants of the LTP learning window are usually smaller than the time constants of the LTD window ([40], [60]), which will further enhance the specificity of the LTP learning as opposed to the LTD part that computes the average over a longer window.

Note that the exponential weight dependence of the learning rule implies a certain robustness towards linearly scaling LTP or LTD strengths, which only leads to a constant offset of the weights. Assuming that the offset is the same for all synapses, this does not affect firing probabilities of neurons in a WTA circuit (see Methods “Weight offsets and positive weights”).

### Example 2: Learning of probabilistic models for orientation selectivity

**Figure 5:**
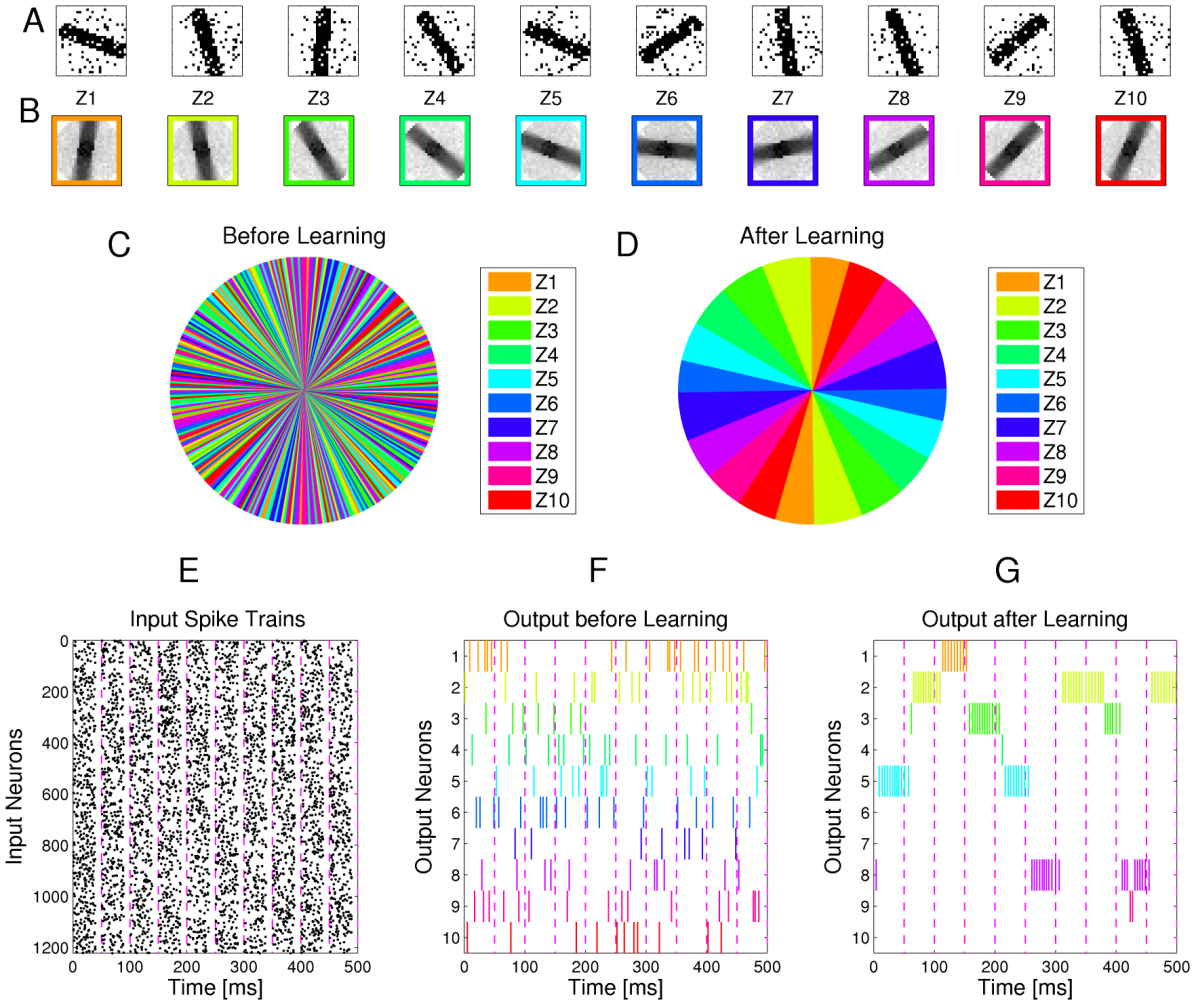
Emergence of orientation selective cells for visual input consisting of oriented bars with random orientations. **A** Examples of 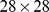-pixel input images with oriented bars and additional background noise. **B** Internal models (weight vectors of output neurons 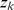) that are learned through STDP after the presentation of 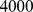 input images (each encoded by spike trains for 50 ms, as in Fig. 3). **C, D** Plot of the most active neuron for 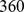 images of bars with orientations from 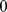 to 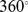 in 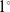 steps. Colors correspond to the colors of 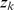 neurons in B. Before training (**C**), the 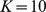 output neurons fire without any apparent pattern. After training (**D**) they specialize on different orientations and cover the range of possible angles approximately uniformly. **E**: Spike train encoding of the 10 samples in A. **F,G**: Spike trains produced by the 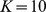 output neurons in response to these samples before and after learning with STDP for 200 s. Colors of the spikes indicate the identity of the output neuron, according to the color code in B.

We demonstrated in this computer experiment the emergence of orientation selective cells 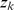 through STDP in the WTA circuit of Fig. 1A when the spike inputs encode isolated bars in arbitrary orientations. Input images were generated by the following process: Orientations were sampled from a uniform distribution, and lines of 7 pixels width were drawn in a 28×28 pixel array. We added noise to the stimuli by flipping every pixel with a 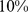 chance, see Fig. 5A. Finally, a circular mask was applied to the images to avoid artifacts from image corners. Spikes trains 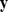 were encoded according to the same population coding principle described in the previous example Fig. 3, in this case using a Poisson firing rate of 20 Hz for active units.

After training with STDP for 200 s, presenting 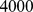 different images, the projection of the learned weight vectors back into the 2D input space (Fig. 5B) shows the emergence of 10 models with different orientations, which cover the possible range of orientations almost uniformly. When we plot the strongest responding neuron as a function of orientation (Fig. 5C, D), measured by the activity in response to 360 noise-free images of oriented bars in 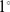 steps, we can see no structure in the response before learning (Fig. 5C). However, after unsupervised learning, panel D clearly shows the emergence of continuous, uniformly spaced regions in which one of the 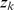 neurons fires predominantly. This can also be seen in the firing behavior in response to the input spike trains in Fig. 5E, which result from the example images in panel A. Fig. 5F shows that the output neurons initially fire randomly in response to the input, and many different 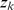 neurons are active for one image. In contrast, the responses after learning in panel G are much sparser, and only occasionally multiple neurons are active for one input image, which is the case when the angle of the input image is in between the preferred angles of two output neurons, and therefore multiple models have a non-zero probability of firing.

In our experiment the visual input consisted of noisy images of isolated bars, which illustrates learning of a probabilistic model in which a continuous hidden cause (the orientation angle) is represented by a population of neurons, and also provides a simple model for the development of orientation selectivity. It has previously been demonstrated that similar Gabor-like receptive field structures can be learned with a sparse-coding approach using patches of natural images as inputs [61]. The scenario considered here is thus substantially simplified, since we do not present natural but isolated stimuli. However, it is worth noting that experimental studies have shown that (in mice and ferret) orientation selectivity, but not e.g. direction selectivity, exists in V1 neurons even before eye opening [62], [63]. This initial orientation selectivity develops from innate mechanisms and from internally generated inputs during this phase [63], e.g. retinal waves, which have different, and very likely simpler statistics than natural stimuli. Our model shows that a WTA circuit could learn orientation selectivity from such simple bar-like inputs, but does not provide an alternative explanation to the results of studies like [61] using natural image stimuli. Although beyond the scope of this paper, we expect that later shaping of selectivity through exposure to natural visual experience would not alter the receptive fields by much, since the neurons have been primed to spike (and thereby trigger plasticity) only in response to a restricted class of local features.

### Example 3: Emergent discrimination of handwritten digits through STDP

**Figure 6:**
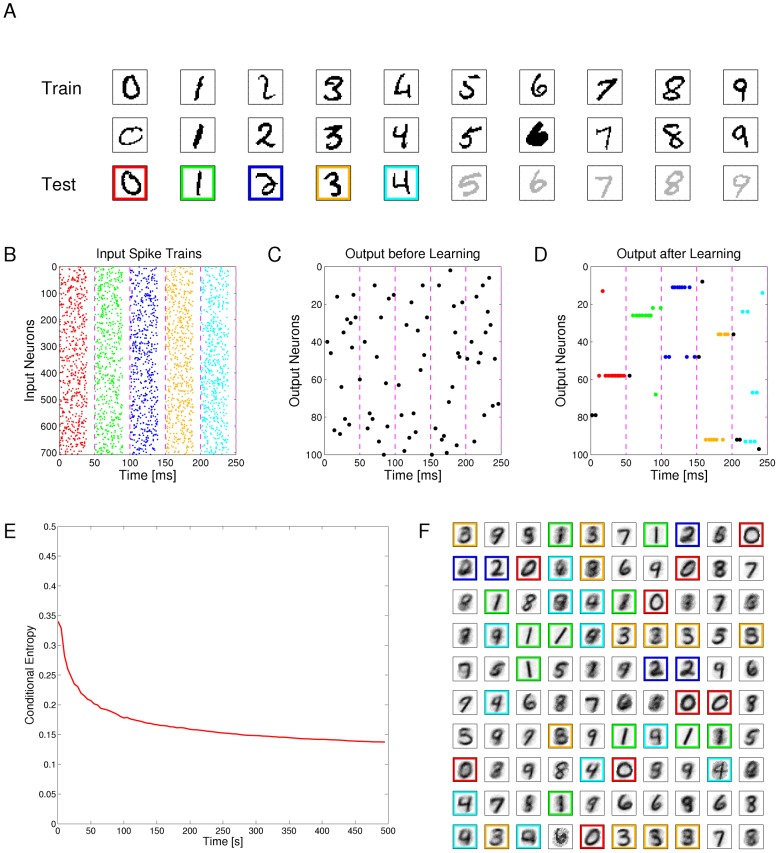
Emergent discrimination of handwritten digits through STDP. **A**: Examples of digits from the MNIST dataset. The third and fourth row contain test examples that had not been shown during learning via STDP. **B**: Spike train encoding of the first 5 samples in the third row of A. Colors illustrate the different classes of digits. **C, D**: Spike trains produced by the 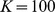 output neurons before and after learning with STDP for 500 s. Colored spikes indicate that the class of the input and the class for which the neuron is mostly selective (based on human classification of its generative model shown in F) agree, otherwise spikes are black. **E**: Temporal evolution of the self-organization process of the 100 output neurons (for the complex version of STDP-curve shown in Fig. 1B), measured by the conditional entropy of digit labels under the learned models at different time points. **F**: Internal models generated by STDP for the 100 output neurons after 500 s. The network had not received any information about the number of different digits that exist and the colors for different ways of writing the first 5 digits were assigned by the human supervisor. On the basis of this assignment the test samples in row 3 of panel A had been recognized correctly.

Spike-based EM is a quite powerful learning principle, as we demonstrate in Fig. 6 through an application to a computational task that is substantially more difficult than previously considered tasks for networks of spiking neurons: We show that a simple network of spiking neurons can learn without any supervision to discriminate handwritten digits from the MNIST benchmark dataset [64] consisting of 70,000 samples (30 are shown in Fig. 6A). This is one of the most frequently used benchmark tasks in machine learning. It has mostly been used to evaluate supervised or semi-supervised machine learning algorithms [27], [65], or to evaluate unsupervised feature learning approaches [66], [67]. Although the MNIST dataset contains labels (the intended digit) for each sample of a handwritten digit, we deleted these labels when presenting the dataset to the neural circuit of Fig. 1A, thereby forcing the 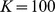 neurons on the output layer to self-organize in a completely unsupervised fashion. Each sample of a handwritten digit was encoded by 708 spike trains over 40 ms (and 10 ms periods without firing between digits to avoid overlap of EPSPs between images), similarly as for the task of Fig. 3. Each pixel was represented by two input neurons 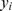, one of which produced a Poisson spike train at 40 Hz during these 40 ms. This yielded usually at most one or two spikes during this time window, demonstrating that the network learns and computes with information that is encoded through spikes, rather than firing rates. After 500 s of unsupervised learning by STDP almost all of the output neurons fired more sparsely, and primarily for handwritten samples of just one of the digits (see Fig. 6E).

The application to the MNIST dataset had been chosen to illustrate the power of SEM in complex tasks. MNIST is one of the most popular benchmarks in machine learning, and state-of-the-art methods achieve classification error rates well below 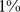. The model learned by SEM can in principle also be used for classification, by assigning each neuron to the class for which it fires most strongly. However, since this is an unsupervised method, not optimized for classification but for learning a generative model, the performance is necessarily worse. We achieve an error rate of 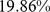 on the 10-digit task on a previously unseen test set. This compares favorably to the 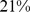 error that we obtained with a standard machine learning approach that directly learned the mixture-of-multinomials graphical model in Fig. 1B with a batch EM algorithm. This control experiment was not constrained by a neural network architecture or biologically plausible learning, but instead mathematically optimized the parameters of the model in up to 200 iterations over the whole training set. The batch method achieves a final conditional entropy of 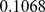, which is slightly better than the 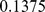 final result of the SEM approach, and shows that better performance on the classification task does not necessarily mean better unsupervised model learning.

### Example 4: Detection of Spatio-Temporal Spike Patterns

**Figure 7:**
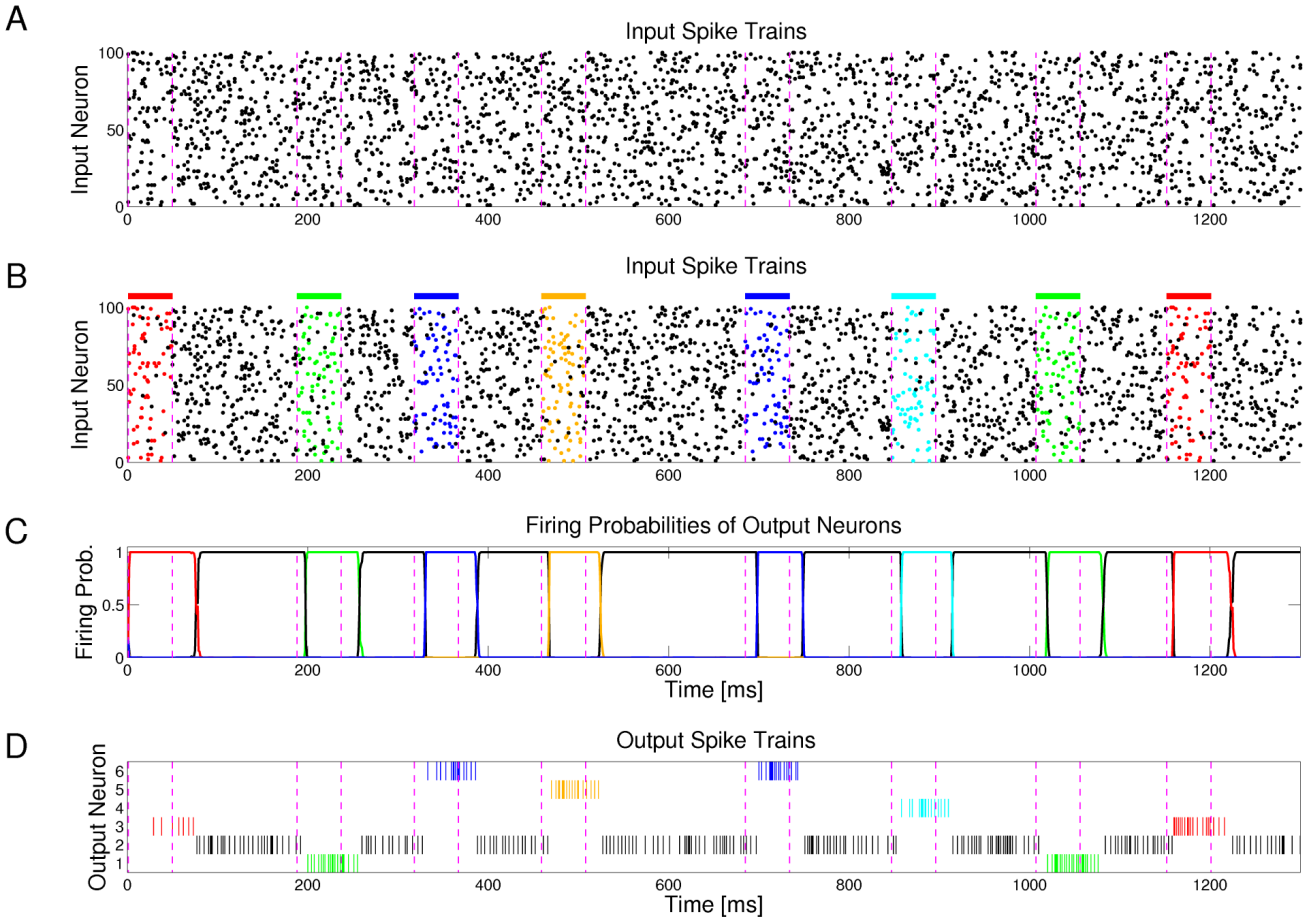
Output neurons self-organize via STDP to detect and represent spatio-temporal spike patterns. **A**: Sample of the Poisson input spike trains at 20 Hz (only 100 of the 500 input channels are shown). Dashed vertical lines mark time segments of 50 ms length where spatio-temporal spike patterns are embedded into noise. **B**: Same spike input as in A, but spikes belonging to five repeating spatio-temporal patterns (frozen Poisson spike patterns at 15 Hz) are marked in five different colors. These spike patterns are superimposed by noise (Poisson spike trains at 5 Hz), and interrupted by segments of pure noise of the same statistics (Poisson spike trains at 20 Hz) for intervals of randomly varying time lengths. **C, D**: Firing probabilities and spike outputs of 6 output neurons (z-neurons in Fig. 1A) for the spike input shown in A, after applying STDP for 200 s to continuous spike trains of the same structure (without any supervision or reward). These 6 output neurons have self-organized so that 5 of them specialize on one of the 5 spatio-temporal patterns. One of the 6 output neurons (firing probability and spikes marked in black) only responds to the noise between these patterns. The spike trains in A represent test inputs, that had never been shown during learning.

Our final application demonstrates that the modules for Bayesian computation that emerge in WTA circuits through STDP can not only explain the emergence of feature maps in primary sensory cortices like in Fig. 5, but could also be viewed as generic computational units in generic microcircuits throughout the cortex. Such generic microcircuit receives spike inputs from many sources, and it would provide a very useful computational operation on these if it could autonomously detect repeatedly occurring spatio-temporal patterns within this high-dimensional input stream, and report their occurrence through a self-organizing sparse coding scheme to other microcircuits. We have created such input streams with occasionally repeated embedded spike patterns for the computer experiment reported in Fig. 7. Fig. 7D demonstrates that sparse output codes for the 5 embedded spike patterns emerge after applying STDP in a WTA circuit for 200 s to such input stream. Furthermore, we show in the Supplement that these sparse output codes generalize (even without any further training) to time-warped versions of these spike patterns.

Even though our underlying probabilistic generative model (Fig. 1B) does not include time-dependent terms, the circuit in this example performs inference over time. The reason for this is that synapses that were active when a neuron fired become reinforced by STDP, and therefore make the neuron more likely to fire again when a similar spatial pattern is observed. Since we use EPSPs that smoothly decay over time, one neuron still sees a trace of previous input spikes as it fires again, and thus different spatial patterns within one reoccurring spatio-temporal pattern are recognized by the same neuron. The maximum length for such patterns is determined by the time constants of EPSPs. With our parameters (1 ms rise, 15 ms decay time constant) we were able to recognize spike patterns up to 50–100 ms. For longer spatio-temporal patterns, different neurons become responsive to different parts of the pattern. The neuron that responds mostly to noise in Figs. 7D did not learn a specific spatial pattern, and therefore wins by default when none of the specialized neurons responds. Similar effects have previously been described [59], [68], but for different neuron models, classical STDP curves, and not in the context of probabilistic inference.

For this kind of task, where also the exact timing of spikes in the patterns matters (which is not necessarily the case in the examples in Figs. 3, 5, and 6, where input neurons generate Poisson spike trains with different rates), we found that the shape of the STDP kernel plays a larger role. For example, a time-inverted version of the SEM rule, where pre-before-post firing causes LTD instead of LTP, cannot learn this kind of task, because once a neuron has learned to fire for a sub-pattern of the input, its firing onset is shifted back in time, rather than forward in time, which happens with standard SEM, but also with classical STDP [50], [59]. Instead, with a time-inverted SEM rule, different neurons would learn to fire stronger for the offsets of different patterns.

Such emergent compression of high-dimensional spike inputs into sparse low-dimensional spike outputs could be used to merge information from multiple sensory modalities, as well as from internal sources (memory, predictions, expectations, etc.), and to report the co-occurrence of salient events to multiple other brain areas. This operation would be useful from the computational perspective no matter in which cortical area it is carried out. Furthermore, the computational modules that we have analyzed can easily be connected to form networks of such modules, since their outputs are encoded in the same way as their inputs: through probabilistic spiking populations that encode for abstract multinomial variables. Hence the principles for the emergence of Bayesian computation in local microcircuits that we have exhibited could potentially also explain the self-organization of distributed computations in large networks of such microcircuits.

## Discussion

We have shown that STDP induces a powerful unsupervised learning principle in networks of spiking neurons with lateral inhibition: spike-based Expectation Maximization. Each application of STDP can be seen as a move in the direction of the M-step in a stochastic online EM algorithm that strives to maximize the log-likelihood 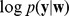 of the spike input 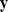. This is equivalent to the minimization of the Kullback-Leibler divergence between the true distribution 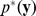 of spike inputs, and the generative model 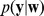 that is implicitly represented by the WTA circuit from the Bayesian perspective. This theoretically founded principle guarantees that iterative applications of STDP to different spike inputs do not induce a meaningless meandering of the synaptic weights 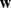 through weight space, but rather convergence to at least a local optimum in the fitting of the model to the distribution 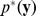 of high-dimensional spike inputs 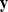. This generation of an internal model through STDP provides the primary component for the self-organization of Bayesian computation. We have shown that the other component, the prior, results from a simple rule for use-dependent adaptation of neuronal excitability. As a consequence, the firing of a neuron 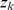 in a stochastic WTA circuit (Fig. 1A) can be viewed as sampling from the posterior distribution of hidden causes for high-dimensional spike inputs 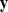 (and simultaneously as the 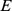-step in the context of online EM): A prior (encoded by the thresholds 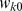 of the neurons 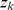) is multiplied with a likelihood (encoded through an implicit generative distribution defined by the weights 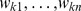 of these neurons 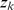), to yield through the firing probabilities of the neurons 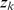 a representation of the posterior distribution of hidden causes for the current spike input 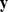. The multiplications and the divisive normalization that are necessary for this model are carried out by the linear neurons in the log-scale. This result is then transformed into an instantaneous firing rate, assuming an exponential relationship between rate and the membrane potential [38]. It is important that the neurons 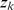 fire stochastically, i.e., that there exists substantial trial-to trial variability, since otherwise they could not represent a probability distribution. Altogether our models supports the view that probability distributions, rather than deterministic neural codes, are the primary units of information in the brain, and that computational operations are carried out on probabilities, rather than on deterministic bits of information.

Following the “probabilistic turn” in cognitive science [3], [4], [69] and related hypotheses in computational neuroscience [1], [2], [5], probabilistic inference has become very successful in explaining behavioral data on human reasoning and other brain functions. Yet, it has remained an important open problem how networks of spiking neurons can learn to implement those probabilistic inference operations and probabilistic data structures. The soft WTA model presented in this article provides an answer for the case of Bayesian inference and learning in a simple graphical model, where a single hidden cause has to be inferred from bottom-up input. Although this is not yet a mechanism for learning to perform general Bayesian inference in arbitrary graphical models, it clearly is a first step into that direction. Importantly, the encoding of posterior distributions through spiking activity of the neurons 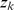 in a WTA circuit is perfectly compatible with the assumed input encoding from external variables 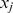 into spiking activity in 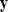. Thus, the interpretation of spikes from output neurons 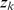 as samples of the posterior distributions over hidden variables in principle allows for using these spikes as input for performing further probabilistic inference.

This compatibility of input and output codes means that SEM modules could potentially be hierarchically and/or recurrently coupled in order to serve as inputs of one another, although it remains to be shown how this coupling affects the dynamics of learning and inference. Future research will therefore address the important questions whether interconnected networks of modules for Bayesian computation that emerge through STDP can provide the primitive building blocks for probabilistic models of cortical computation. Previous studies [23], [25] have shown that interconnected networks of WTA modules are indeed computationally very powerful. In particular, [24], [25] have recently shown how recurrently connected neurons can be designed to perform neural sampling, an approach in which time-independent probability distributions can be represented through spiking activity in recurrent neural networks. The question how salient random variables come to be represented by the firing activity of neurons has remained open. This paper shows that such representations may emerge autonomously through STDP.

A prediction for networks of hierarchically coupled SEM modules would be that more and more abstract hidden causes can be learned in higher layers such as it has been demonstrated in machine learning approaches using Deep Belief Networks [66] and more recently in Deep Boltzmann Machines (DBM) [70]. This effect would correspond to the emergence of abstract feature selectivity in higher visual areas of primates (e.g. face-selective cells in IT, [71]). The hierarchical structure, however, that would result from such deeply organized SEM-modules is more reminiscent of a Deep Sum-Product Network [72], a recently presented new architecture, which has a much simpler learning dynamics but arguably a similar expressive power as DBM. In addition, with a consistent input encoding, associations between different sensory modalities could be formed by connecting inputs from different low-level or high-level sources to a single SEM.

Importantly, while the discussion above focused only on the representation of complex stimuli by neurons encoding abstract hidden causes, SEM can also be an important mechanism for fast and reliable reinforcement learning or decision making under uncertainty. Preprocessing via single or multiple SEM circuits provides an abstraction of the state of the organism, which is much lower-dimensional than the complete stream of individual sensory signals. Learning a behavioral strategy by reading out such behaviorally relevant high-level state signals and mapping them into actions could therefore speed up learning by reducing the state space. In previous studies [21], [73] we have shown how optimal strategies can be learned very fast by simple local learning rules for reinforcement learning or categorization, if a preprocessing of input signals based on probabilistic dependencies is performed. SEM would be a suitable unsupervised mechanism for learning such preprocessing networks for decision making.

We also have shown that SEM is a very powerful principle that endows networks of spiking neurons to solve complex tasks of practical relevance (see e.g. Fig. 6), and as we have shown, their unsupervised learning performance is within the range of conventional machine learning approaches. Furthermore, this could be demonstrated for computations on spike inputs with an input dimension of about 1000 presynaptic neurons 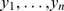, a number that approaches the typical dimension of the spike input that a cortical neuron receives. A very satisfactory aspect is that this high computational performance can be achieved by networks of spiking neurons that learn completely autonomously by STDP, without any postulated teacher or other guidance. This could benefit the field of neuromorphic engineering [74]–[76], which develops dedicated massively parallel and very efficient hardware for emulating spiking neural networks and suitable plasticity rules. The link between spiking neuron models and plasticity rules and established machine learning concepts provides a novel way of installing well-understood Bayesian inference and learning mechanisms on neuromorphic hardware. First steps towards implementing SEM-like rules in different types of neuromorphic hardware have been taken.

### Prior related work

A first model for competitive Hebbian learning paradigm in non-spiking networks of neurons had been introduced in [77]. They analyzed a Hebbian learning rule in a hard WTA network and showed that there may exist equilibrium states, in which the average change of all weight values vanishes for a given set of input patterns. They showed that in these cases the weights adopt values that are proportional to the conditional probability of the presynaptic neuron being active given that the postsynaptic unit wins (rather than the log of this conditional probability, as in our framework). [78] showed that the use of a soft competition instead of a hard winner assignment and corresponding average weight updates lead to an exact gradient ascent on the log-likelihood function of a generative model of a mixture of Gaussians. However, these learning rules had not yet been analyzed in the context of EM.

Stochastic approximation algorithms for expectation maximization [31] were first considered in [79], incremental and on-line EM algorithms with soft-max competition in [80]–[82]. A proof of the stochastic approximation convergence for on-line EM in exponential family models with hidden variables was shown in [29]. They developed a sophisticated schedule for the learning rate in this much more general model, but did not yet consider individual learning rates for different weights.

[54] initiated the investigation of STDP in the context of unsupervised competitive Hebbian learning and demonstrated that correlations of input spike trains can be learned in this way. They also showed that this leads to a competition between the synapses for the control of the timing of the postsynaptic action potential. A similar competition can also be observed during learning in our model, since our learning rule automatically drives the weights towards satisfying the normalization conditions in Eq. (10).

[83] present a network and learning model that is designed to perform Independent Component Analysis (ICA) with spiking neurons through STDP and intrinsic plasticity. The mixture model of independent components can also be formulated as a generative model, and the goal of ICA is to find the optimal parameters of the mixing matrix. It has been shown that also this problem can be solved by a variant of Expectation Maximization [84], so there is some similarity to the identification of hidden causes in our model.

Recently, computer experiments in [85], [86] have used STDP in the context of WTA circuits to achieve a clustering of input patterns. Their STDP rules implements linear updates, independent of the current weight values, mixed with a homeostasis rule to keep the sum of all weights constant and every weight between 0 and 1. This leads to weights that are roughly proportional to the probability of the presynaptic neuron's firing given that the post-synaptic neuron fires afterwards. The competition between the output neurons is carried out as hard-max. In [85] the 4 output neurons learn to differentiate the 4 presented patterns and smoothly interpolate new rotated input patterns, whereas in [86] 48 neurons learn to differentiate characters in a small pixel raster. [86] uses a STDP rule where both LTP and LTD are modeled as exponentially dependent on the time difference. However, the very specific experimental setting with synchronous regular firing of the input neurons makes it difficult to generalize their result to more general input spike trains. No theoretical analysis is provided in [85] or [86], but their experimental results can be explained by our SEM approach. Instead of adding up logs of conditional probabilities and performing the competition on the exponential of the sums, they sum up the conditional probabilities directly and use this sum of probabilities for the competition. This can be seen as a linear approximation of SEM, especially under the additional normalization conditions that they impose by homeostasis rules.

It has previously been shown that spike patterns embedded in noise can be detected by STDP [49], [50], [59]. Competitive pattern learning through STDP has recently been studied in [68]. They simulate a deterministic version of a winner-take-all circuit consisting of a fixed number of neurons, all listening to the same spiking input lines and connected to each other with a strong inhibition. The STDP learning rule that they propose is additive and weight-independent. Just like our results, they also observe that different neurons specialize on different fixed repeated input pattern, even though the repeated patterns are embedded in spiking noise such that the mean activity of all inputs remains the same throughout the learning phase. Additionally they show that within each pattern the responsible neuron tries to detect the start of the pattern. In contrast to our approach they do not give any analysis of convergence guarantees, nor does their model try to build a generative probabilistic model of the input distribution.

[87]–[89] investigated the possibility to carry out Bayesian probabilistic computations in recurrent networks of spiking neurons, both using probabilistic population codes. They showed that the ongoing dynamics of belief propagation in temporal Bayesian models can be represented and inferred by such networks, but they do not exhibit any neuronal plausible learning mechanism. [7] presented another approach to Bayesian inference using probabilistic population codes, also without any learning result.

An interesting complementary approach is presented in [90], [91], where a single neuron is modeled as hidden Markov model with two possible states. This approach has the advantage, that the instantaneous synaptic input does not immediately decide the output state, but only incrementally influences the probability for switching the state. The weights and the temporal behavior can be learned online using local statistics. The downside of this approach is that this hidden Markov model can have only two states. In contrast, the SEM approach can be applied to networks with any number of output neurons.

In [92] it was shown that a suitable rule for supervised spike-based learning (the Tempotron learning rule) can be used to train a network to recognize spatio-temporal spike patterns. This discriminative learning scheme enables the recognizing neuron to focus on the most discriminative segment of the pattern. In contrast, our generative unsupervised learning scheme drives the recognizing neuron to generalize and spike many times during the whole pattern, and thus learns the spatial average activity pattern. The conductance based approach of [92] differs drastically from our method (and the results shown in the Supplement) insofar as here only STDP was used (focusing on average spatial patterns), no supervision was involved, and the time-warped input pattern had never been shown during training.

An alternative approach to implement the learning of generative probabilistic models in spiking neuronal networks is given in [93], [94]. Both approaches are based on the idea to model a sequence of spikes in a Hidden-Markov-Model-like probabilistic model and learn the model parameters through different variants of EM, in which a sequence of spikes represents one single sample of the model's distribution. Due to the explicit incorporation of inference over time, these models are more powerful than ours and thus require non-trivial, non-local learning mechanisms.

### Experimentally testable predictions of the proposed model

**Figure 8:**
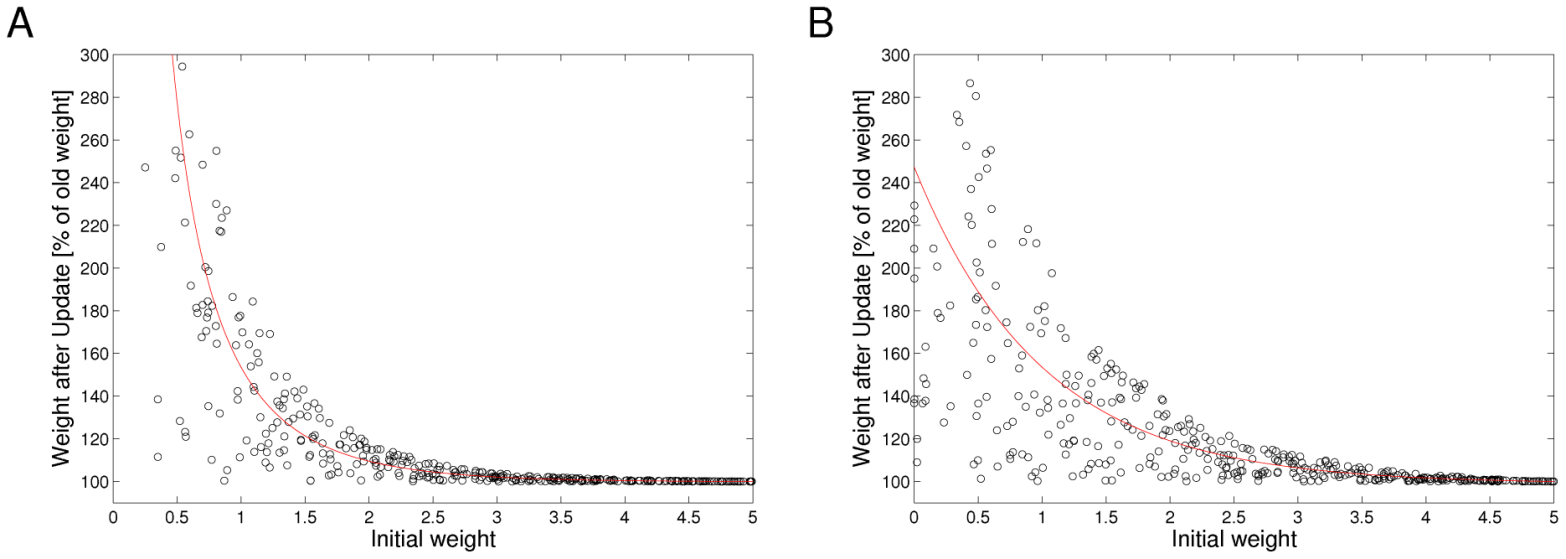
Ideal dependence of weight potentiation under STDP on the initial value of the weight (solid lines). Open circles represent results of samples from this ideal curve with 100% noise, that can be used in the previously discussed computer experiments with almost no loss in performance. **A**: Dependence of weight potentiation on initial weight according to the STDP rule in Eq. (5). **B**: Same with an additional factor 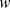.

Our analysis has shown, that STDP supports the creation of internal models and implements spike-based EM if changes of synaptic weights depend in a particular way on the current value of the weight: Weight potentiation depends in an inversely exponential manner on the current weight (see Eq. (5)). This rule for weight potentiation (see Fig. 8A) is consistent with all published data on this dependence: Fig. 5 in [43] and Fig. 5C in [40] for STDP, as well as Fig. 10 in [95] and Fig. 1 in [96] for other protocols for LTP induction. One needs to say, however, that these data exhibit a large trial-to-trial variability, so that it is hard to infer precise quantitative laws from them. On the other hand, the applications of STDP that we have examined in Fig. 3–7 work almost equally well if the actual weight increase varies by up to 100% from the weight increase proposed by our STDP rule (see open circles in Fig. 8A). The resulting distribution of weight increases matches qualitatively the above mentioned experimental data quite well.

The prediction of our model for the dependence of the amount of weight depression on the current weight is drastically different: Even though we make the strong simplification that the depression part of the STDP rule is independent of the time difference between pre- and postsynaptic spike, the formulation in Eq. (5) makes the assumption, that the amount of the depression should be independent of the current weight value. It is this contrast between an exponential dependency for LTP and a constant LTD which makes the weight converge to the logarithm of the conditional presynaptic firing probability in Eq. (6). In experiments this dependency has been investigated in-vitro [40]. There it has been found that the *percentage* of weight depression under STDP is independent of the current weight, which implies that the amount of depression is linear in the current weight value. This seems to contradict the presented learning rule. However, the key property that is needed for the desired equilibrium condition is the ratio between LTP and LTD. So the equilibrium proof in Eq. (28) remains unchanged if 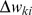 is multiplied (for potentiation and depression) by some arbitrary function 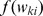 of the current weight value. Choosing for example 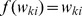 yields a depression whose percentage is independent of the initial value, which would be consistent with the above mentioned in-vitro data [40]. The resulting dependence for potentiation is plotted in Fig. 8B. Since this curve is very similar to that of Fig. 8A, the above mentioned experimental data for potentiation are too noisy to provide a clear vote for one of these two curves. Thus more experimental data are needed for determining the dependence of weight potentiation on the initial weight. Whereas the relevance of this dependency had previously not been noted, our analysis suggests that such a contrast it is in fact essential for the capability of STDP to create internal models for high-dimensional spike inputs.

Our analysis has shown, that if the excitability of neurons is also adaptive, with a rule as in Eq. (7) that is somewhat analogous to that for synaptic plasticity, then neurons can also learn appropriate priors for Bayesian computation. Several experimental studies have already confirmed, that the intrinsic excitability of neurons does in fact increase when they are more frequently activated [45], see [97], [14] and [39] for reviews. But a quantitative study, which relates the resulting change in intrinsic excitability to its initial value, is missing.

Our model proposes that pyramidal neurons in cortical microcircuits are organized into stochastic WTA circuits, that together represent a probability distribution. This organization is achieved by a suitably regulated common inhibitory signal, where the inhibition follows the excitation very closely. Such instantaneous balance between excitation and inhibition was described by [34]. A resulting prediction of the WTA structure is that the firing activity of these neurons is highly de-correlated due to the inhibitory competition. In contrast to previous experimental results, that reported higher correlations, it has recently been confirmed in [35] for the visual cortex of awake monkey that nearby neurons, even though they share common input show extremely low correlations.

Another prediction is that neural firing activity especially for awake animals subject to natural stimuli is quite sparse, since only those neurons fire whose internal model matches their spike input. A number of experimental studies confirm this predictions (see [98] for a review). Our model also predicts, that the neural firing response to stimuli exhibits a fairly high trial-to-trial variability, as is typical for drawing repeated samples from a posterior distribution (unless the posterior probability is close to 0 or 1). A fairly high trial-to-trial variability is a common feature of most recordings of neuronal responses (see e.g. [99], Fig. 1B in [100]; a review is provided in [101]). In addition, our model predicts that this trial-to-trial variability decreases for repeatedly occurring natural stimuli (especially if this occurs during attention) and discrimination capability improves for these stimuli, since the internal models of neurons are becoming better fitted to their spike input during these repetitions (“sharpening of tuning”), yielding posterior probabilities closer to 1 or 0 for these stimuli. These predictions are consistent with a number of experimental data related to perceptual learning [102], [103], and with the evolution of neuronal responses to natural scenes that were shown repeatedly in conjunction with nucleus basalis stimulation [104].

In addition our model predicts that if the distribution of sensory inputs changes, the organization of codes for such sensory inputs also changes. More frequently occurring sensory stimuli will be encoded with a finer resolution (see [105] for a review of related experimental data). Furthermore in the case of sensory deprivation (see [106]) our model predicts that neurons that used to encode stimuli which no longer occur will start to participate in the encoding of other stimuli.

We have shown in Fig. 3 that an underlying background oscillation on neurons that provide input to a WTA circuit speeds up the learning process, and produces more precise responses after learning. This result predicts that cortical areas that collaborate on a common computational task, especially under attention, exhibit some coherence in their LFP. This has already been shown for neurons in close proximity [107] but also for neurons in different cortical areas [108], [109].

If one views the modules for Bayesian computation that we have analyzed in this article as building blocks for larger cortical networks, these networks exhibit a fundamental difference to networks of neurons: Whereas a neuron needs a sufficiently strong excitatory drive in order to reach its firing threshold, the output neurons 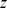 of a stochastic WTA circuit according to our model in Eq. (3) are firing already on their own - even without any excitatory drive from the input neuron 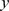 (due to assumed background synaptic inputs; modeled in our simulations by an Ornstein-Uhlenbeck process, as suggested by in-vivo data [110]). Rather, the role of the input from the 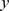-neurons is to modulate which of the neurons in the WTA circuit fire. One consequence of this characteristic feature is that even relatively few presynaptic neurons 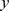 can have a strong impact on the firing of the 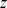-neurons, provided the 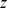-neurons have learned (via STDP) that these 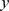-neurons provide salient information about the hidden cause for the total input 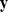 from all presynaptic neurons. This consequence is consistent with the surprisingly weak input from the LGN to area V1 [16], [111], [112]. It is also consistent with the recently found exponential distance rule for the connection strength between cortical areas [112]. This rule implies that the connection strength between distal cortical areas, say between primary visual cortex and PFC, is surprisingly weak. Our model suggests that these weak connections can nevertheless support coherent brain computation and memory traces that are spread out over many, also distal, cortical areas.

Apart from these predictions regarding aspects of brain computation on the microscale and macroscale, a primary prediction of our model is that complex computations in cortical networks of neurons - including very efficient and near optimal processing of uncertain information - are established and maintained through STDP, on the basis of genetically encoded stereotypical connection patterns (WTA circuits) in cortical microcircuits.

## Methods

According to our input model, every external multinomial variable 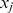, with 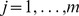 is encoded through a group 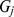 of neurons 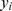, with 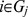. The generative model 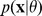 from Fig. 1B is implicitly encoded in the WTA circuit of Fig. 1A with 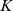 excitatory neurons 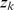 by:
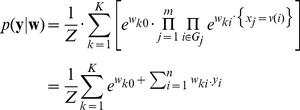
(19)where 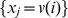 is the binary indicator function of 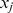 taking on value 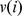. In the generative model 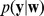 we define the binary variables 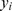 and set 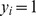 if 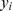 represents the value 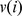 of the multinomial variable 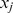 (with 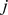 s.t. 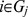) and 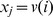, otherwise 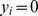. The sets 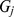 represent a partition of 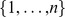, thus 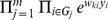 and the form 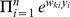 used in Eq. (9) are equivalent expressions. The value of the normalization constant 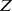 can be calculated explicitly as
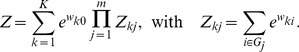
(20)This generative model can be rewritten as a mixture distribution with parameters 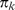 and 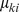:
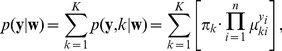
(21)

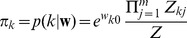
(22)

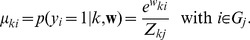
(23)In order to show how the constants 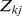 cancel out we write the full joint distribution of 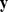 and the “hidden cause” 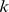 as the product of the prior 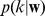 and the likelihood 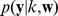:
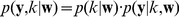
(24)

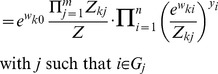
(25)

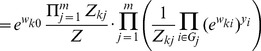
(26)

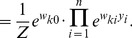
(27)


Under the normalization conditions in Eq. (10) the parameters of the mixture distribution simplify to 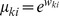 and 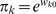, since all 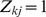 and 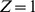.

The generative model in Eq. (24) is well defined only for vectors 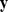, such that there is exactly one “1” entry per group 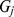. However, in the network model with rectangular, renewable EPSPs, there are time intervals where 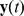 may violate this condition, if the interval between two input spikes is longer that 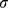. It is obvious from Eq. (24) that this has the effect of dropping all factors representing 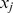, since this results in an exponent of 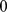. Under proper normalization conditions (or at least if all 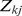 have identical values), this drop of an entire input group in the calculation of the posterior in Eq. (11) is identical to performing inference with unknown 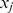 (see ‘Impact of missing input values’). Eq. (11) holds aslong as there are no two input spikes from different neurons within the same group closer than 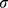, which we have assumed for the simple input model with rectangular, renewable EPSPs.

### Equilibrium condition

We will now show that all equilibria of the stochastic update rule in Eq. (5) and Eq. (7), i.e., all points where 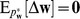, exactly match the implicit solution conditions in Eq. (46), and vice versa:
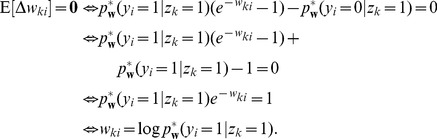
(28)Analogously, one can show that 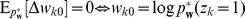. Note that this result implies that the learning rule in Eq. (5) and Eq. (7) has no equilibrium points outside the normalization conditions in Eq. (10), since all equilibrium points fulfill the implicit solutions condition in Eq. (46) and these in turn fulfill the normalization conditions.

### Details to *Learning the parameters of the probability model by EM*

In this section we will analyze the theoretical basis for learning the parameters 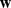 of the generative probability model 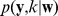 given in Eq. (9) from a machine learning perspective. In contrast to the intuitive explanation of the Results section which was based on Expectation Maximization we will now derive an implicit analytical solution for a (locally) optimal weight vector 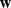, and rewrite this solution in terms of log probabilities. We will later use this derivation in order to show that the stochastic online learning rule provably converges towards this solution.

For an exact definition of the learning problem, we assume that the input is given by a stream of vectors 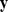, in which every 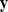 is drawn independently from the input distribution 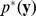. In principle, this stream of 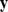
's corresponds to the samples 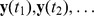 that are observed at the spike times 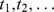 of the circuit. However, in order to simplify the proofs in this and subsequent sections, we will neglect any possible temporal correlation between successive samples.

The learning task is to find parameter values 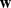, such that the marginal 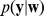 of the model distribution 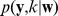 approximates the actual input distribution 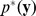 as accurately as possible. This is equivalent to minimizing the Kullback-Leibler divergence between the two distributions:
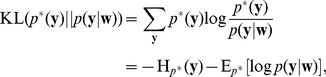
(29)where 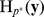 is the (constant) entropy of the input distribution 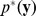, and 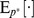 denotes the expectation over 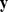, according to the distribution 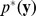. Since 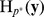 is constant, minimizing the right hand side of Eq. (29) is equivalent to maximizing the expected log likelihood 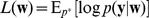.

There are many different parametrizations 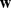 that define identical generative distributions 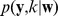 in Eq. (24). There is, however, exactly one 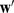 in this sub-manifold of the weight space that fulfills the normalization conditions in Eq. (10).

We thus redefine the goal of learning more precisely as the constrained maximization problem
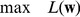
(30)

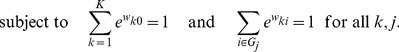
(31)


This maximization problem never has a unique solution 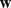, because any permutation of the values of 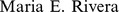 and their corresponding weights leads to different joint distributions 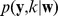, all of them having identical marginals 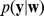. The local maxima of Eq. (30) can be found using the Lagrange multiplier method.

Note that we do at no time enforce normalization of 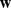 during the learning process, nor do we require normalized initialization of 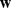. Instead, we will show that the learning rule in Eq. (5,7) automatically drives 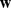 towards a local maximum, in which the normalization conditions are fulfilled.

Under the constraints in Eq. (31) the normalization constant 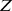 in Eq. (21) equals 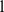, thus 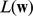 simplifies to 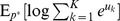 - with 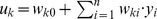 - and we can define a Lagrangian function 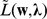 for the maximization problem in Eq. (30,31) by
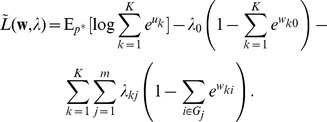
(32)Setting the derivatives to zero we arrive at the following set of equations in 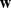 and 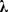:
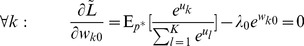
(33)

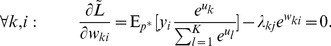
(34)Summing over those equations that have the same multiplier 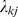 or 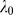, resp., leads to
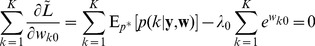
(35)

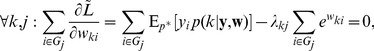
(36)where 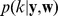 is the shorthand notation for the equivalent expression 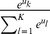. The identity 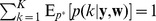, the identity 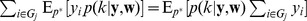 the fact that 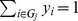, which follows from the definition of population encoding, and the constraints in Eq. (31) are used in order to derive the explicit solution for the Lagrange multipliers
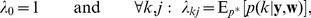
(37)in dependence of 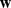. We insert this solution for 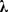 into the gradient Eq. (33,34) and get
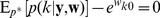
(38)

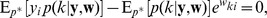
from which we derive an implicit solution for 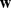:
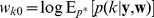
(39)

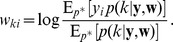
It is easily verified that all fixed points of this implicit solution satisfy the normalization constraints:
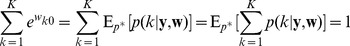
(40)

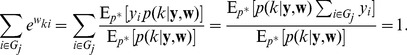
(41)


Finally, in order to simplify the notation we use the augmented input distribution 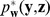. The expectations in Eq. (39) nicely evaluate to
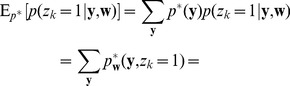
(42)

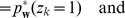
(43)

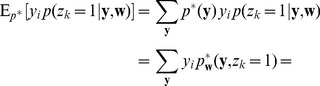
(44)

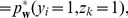
(45)which allows us to rewrite the implicit solution in a very intuitive form as:
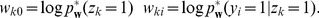
(46)Any weight vector 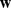 that fulfills Eq. (46) is either a (local) maximum, a saddle point or a (local) minimum of the log likelihood function 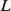 under the normalization constraints.

An obvious numerical approach to solve this fixed point equation is the repeated application of Eq. (39). According to the derivations in the Results section this corresponds exactly to the Expectation Maximization algorithm. But every single iteration asks for the evaluation of expectations with respect to the input distribution 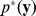, which theoretically requires infinite time in an online learning setup.

### Details to *Spike-based Expectation Maximization*

We derive the update rule in Eq. (5) from the statistical perspective that each weight can be interpreted as 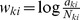, where 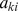 and 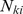 correspond to counters of the events 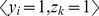 and 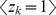. Every new event 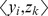 leads to a weight update
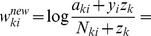
(47)

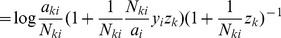
(48)

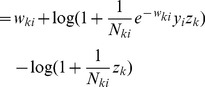
(49)

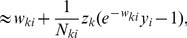
(50)where the log-function is linearly approximated around 1 as 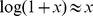. The factor 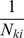 is understood as learning rate 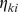 in the additive update rule 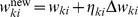. If 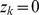, i.e. if there is no postsynaptic spike, the update 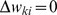. In the case of a postsynaptic spike, i.e., 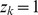, the update 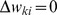 decomposes in the two cases 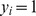 and 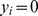 as it is stated explicit in Eq. (5).

As a side note, we observe that by viewing our STDP rule as an approximation to counting statistics, the learning rate 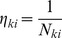 can be understood as the inverse of the equivalent sample size from which the statistics was gathered. If the above rule is used with a small constant learning rate we will get a close approximation to an exponentially decaying average. If the learning rate decays like 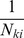 we will get an approximation to an online updated average, where all samples are equally weighted. We will come back to a regulation mechanism for the learning rate in the section ‘Variance Tracking’.

### Details to *Proof of convergence*

In this section we give the proof of Theorem 1. Formally, we define the sequences 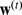, 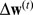, 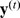, 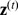 and 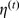 for 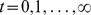: For all 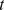 we assume that 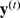 is drawn independently from 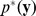. The value of 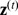 is drawn from the posterior distribution of the model 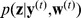 (see Eq. (11)), given the input 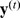 and the current model parameters 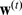. The weight updates 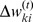, and 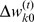, are calculated according to Eq. (5) and (7) with 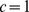. The sequence of weight vectors 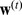 is determined by the randomly initialized vector 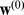, and by the iteration equation
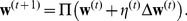
(51)The projection function 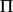 represents a coordinate-wise clipping of 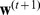 to a hyper-rectangle 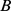 such that 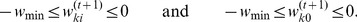
(52)The bound 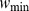 is assumed to be chosen so that all (finite) maxima of 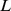 are inside of 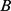. For the sequence of learning rates 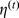 we assume that
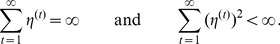
(53)


Under these assumptions we can now restate the theorem formally:

Theorem 1: *The sequence* 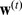 *converges with probability 1 to the set* 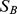 *of all points within the hyper-rectangle* 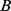 *that fulfill the equilibrium conditions in* *Eq. (6)* *. The stable convergence points among* 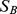 *are the (local) maxima of* 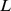 *, subject to the normalization constraints in* *Eq. (10)*.

The iterative application of the learning rule in Eq. (5) and (7) is indeed a stochastic approximation algorithm for learning a (locally) optimal parameter vector 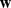. We resort to the theory of stochastic approximation algorithms as presented in [52] and use the method of the “mean limit” ordinary differential equation (ODE). The goal is to show that the sequence of the weight vector 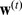 under the stochastic learning rule in Eq. (5) and (7) converges to one of the local maxima of Eq. (30) with probability one, i.e., the probability to observe a non-converging realization of this sequence is zero. The location of the local maximum to which a single sequence of 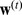 converges depends on the starting point 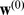 as well as on the concrete realization of the stochastic noise sequence. We will not discuss the effect of this stochasticity in more detail, except for stating that a stochastic approximation algorithm is usually less prone to get stuck in small local maxima than its deterministic version. The stochastic noise introduces perturbations that decrease slowly over time, which has an effect that is comparable to simulated annealing.

We will use the basic convergence theorem of [52] to establish the convergence of the sequence 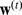 to the limit set of the mean limit ODE. Then it remains to show that this limit set is identical to the desired set of all equilibrium points and thus, particularly, does not contain limit cycles.

**Proof:** In the notation of [52], the mean update of the stochastic algorithm in Eq. (51) is 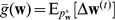. The bounds 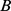 imply that 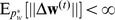 for all 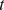 and 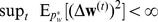.

For any set 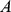 we define 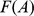 as the positive limit set of the mean limit ODE 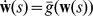 for all initial conditions 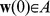:
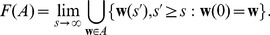
(54)


According to Theorem 3.1 in Chapter 5 of [52], the sequence 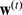 under the algorithm in Eq. (51) converges for all start conditions 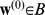 to the limit set 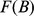 with probability one in the sense that
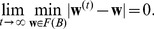
(55)


We will now show that the limit set 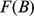 of 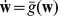 is identical to the set of stationary points 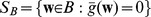 and does not contain limit cycles. It is obvious that 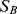 is a subset of 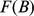 since for all initial conditions 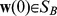 the trajectory of 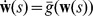 fulfills 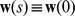 for all 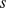. Thus it remains to be shown that there are no other points in 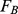 (like e.g. limit cycles).

We split the argument into two parts. In the first part we will show that for 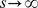 all trajectories of 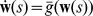 converge asymptotically to the manifold 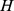 defined by the normalization constraints 31. This leads to the conclusion that 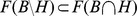. In the second part we will show that all trajectories within 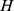 converge to the stationary points 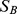, i.e., 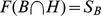. Both parts together yield the desired result that 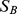 are the only limit points of the ODE 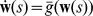.

The first part we start by defining the set of functions 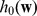 and 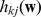 for all 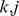 to represent the deviation of the current 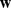 from each of the normalization constraints 31, i.e.,
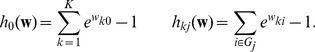
(56)The manifold 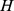 is the set of all points 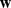 where 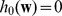 and 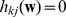 for all 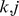. Furthermore, we calculate the gradient vectors 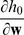 and 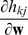 for each of these functions with respect to the argument 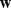. Note that many entries of these gradient vectors are 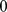, since every single function 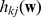 and 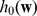 only depends on a few entries of its argument 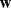. The nonzero entries of these gradients are
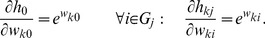
(57)We can now show that the trajectory of 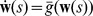 in any point 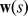 always points in direction of decreasing absolute values for all deviations 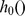 and 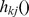:
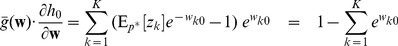
(58)

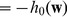
(59)

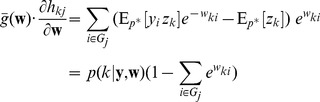
(60)

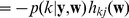
(61)This shows that 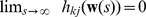 for all 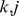 and 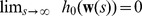. This implies that the limit set of all trajectories with initial conditions outside 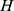 is contained in 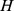, or more formally 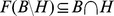. Note that the continuity and the boundedness of 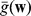 on 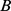 implies 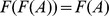 and 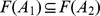 if 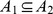 for all 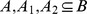. Therefore we can now conclude as the result of the first part
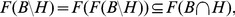
(62)i.e. the limit set of all trajectories starting outside the manifold of normalized weights is contained in the limit set of all trajectories starting within the normalization constraints. The equations (61) also prove that any trajectory with initial condition 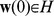 stays within 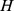, since all components of 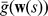 with directions orthogonal to the tangent space of 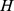 in 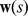 are 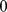 for all 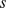, thus 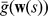 is in the tangent space 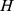 in 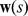.

This immediately leads to the second part of the proof, which is based on the gradient 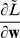 of the Lagrangian 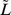 as given in Eq. (33, 34). For any 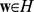 let 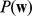 be the linear projection matrix that orthogonally projects any vector 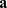 into the tangent space of 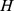 in 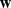. The projection 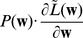 of the gradient of 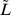 at any 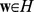 points towards the strongest increase of the value of the objective function 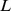 under the constraints of the normalization conditions. Thus, the value of 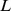 increases in the direction of any vector within the tangent space of 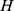 in 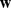 that has a positive scalar product with 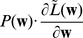. As 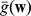 is a tangent vector of 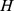 in 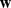 for all 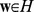, the orthogonal component 
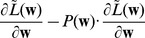
 of the gradient is orthogonal to 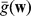. Thus, the value of the scalar product with the projected gradient 
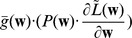
 is identical to the value of the scalar product with the gradient itself 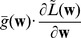:
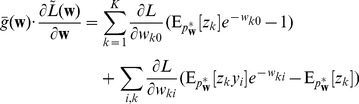
(63)

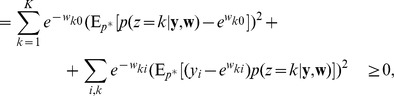
with equality if and only if 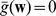, which is equivalent to 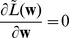. This shows that all trajectories with initial condition 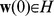 stay within 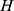 forever and converge to the set of stationary points 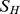, i.e. 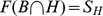. Combining the results of both parts as
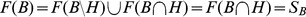
(64)establishes the stochastic convergences of any sequence 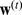 to the set 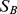 with probability one.

#### Weight offsets and positive weights

All weights 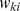 in the theoretical model are logs of probabilities and therefore always have negative values. Through a simple transformation we can shift all weights into the positive range in order to be able to use positive weights only, which is the common assumption for excitatory connections in biologically inspired neural network models. We will now show that setting the parameter 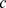 in Eq. (5) different from 1 leads to a linear shift of the resulting weight values by 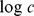, without changing the functionality of the Spike-based EM algorithm.

Firstly, we observe that the application of the update rule in Eq. (5) with 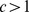 on a shifted weight 
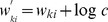
 is identical to the application of the update rule with 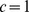 on the original weight 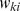, since
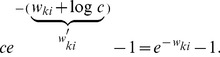
(65)Secondly, we see that the relative firing rate 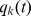 of neuron 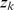 remains unchanged if all weights are subject to the same offset 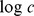, since
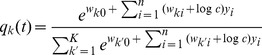
(66)

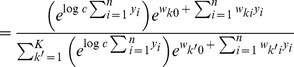
(67)

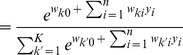
(68)In contrast, the overall firing rate 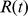 increases by the factor 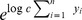. By our definition of the population coding for 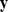, this factor equals 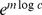, where 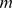 is the number of original input variables 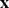. An increase of the inhibitory signal 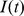 by 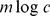 can therefore compensate the increase of overall firing rate. Using this shifted representation, a single excitatory synapse can take on values in the range 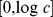, corresponding to probabilities in the range 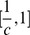.

Similarly the consideration holds valid that it is mathematically equivalent whether the depression of the excitability 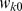 in Eq. (7) is modeled either as an effect of lateral spiking activity or as a constant decay, independent of the circuit activity. In the first case, 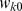 converges to the relative spiking probability of the 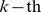 neuron such that the sum of all 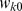 is indeed 1 as described by our theory. In the second case, the 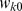 really describe absolute firing rates in some time scale defined by the decay constant. In the logarithmic scale of 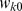 this is nothing else than a constant offset and thus cancels down in Eq. (68).

#### Impact of missing input values

The proof of theorem 1 assumes that every sample 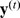 gathered online is a binary vector which contains exactly one entry with value 1 in every group 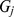. This value indicates the value of the abstract variable 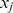 that is encoded by this group. As long as the spikes from the input neurons are closely enough in time, this condition will be fulfilled for every activation vector 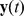. For the cases in which the value of the abstract variable 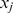 changes, the first spike from group 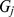 has to appear exactly at that point in time at which the rectangular EPSP for the previous value vanishes, i.e., 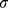 ms after the last preceding spike.

We will now break up this strong restriction of the provable theory and analyze the results that are to be expected, if we allow for interspike intervals longer than 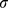. We interpret the resulting “gaps” in the information about the value of an input group as missing value in the sense of Bayesian inference.

We had already addressed the issue of such missing values, resulting from presynaptic neurons that do not spike within the integration time window of an output neuron 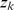, in the discussion of Fig. 3.

A profound analysis of the correct handling of missing data in EM can be found in [48]. Their analysis implies that the correct learning action would be to leave all weights 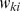 in the group 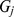 unchanged, if the value of the external variable 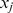 is missing, i.e., if all corresponding 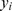
's are 0. However, in this case the STDP rule in Eq. (5) reduces these weights by 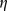. This leads to a modification of the analysis of the equilibrium condition (28):
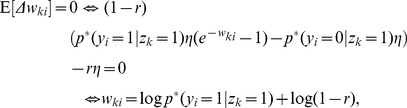
(69)where 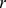 is the probability that 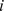 belongs to a group 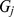 in which the value of 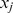 is unknown. We assume that the probability for such a missing value event is independent of the (true) value of the abstract variable 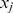 and we assume further that the probability of such missing value events is the same for all groups 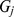 and thus conclude that this offset of 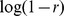 is expected to be the same for all weights. It can easily be verified, that such an offset does not change the resulting probabilities of the competition in the inference according to Eq. (68).

#### Adaptive learning rates with Variance Tracking

In our experiments we used an adaptation of the variance tracking heuristic from [73] for an adaptive control of learning rates. If we assume that the consecutive values of the weights represent independent samples of their true stochastic distribution at the current learning rate, then this observed distribution is the log of a beta-distribution defined by the parameters 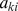 and 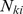 that were used in Eq. (50) to define the update of 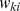 from sufficient statistics. Analytically (see supplement) this distribution has the first and second moments
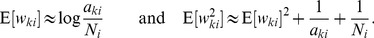
(70)From the first equation we estimate 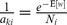. This leads to a heuristic estimate for the (inverse of the) current sample size based on the empirically observed variance 
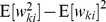
:
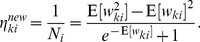
(71)The empirical estimates of these first two moments can be gathered online by exponentially decaying averages using the same learning rate 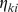. Even though the assumption of independent samples for the estimates of the moments is not met, one can argue about two cases: In case of a stationary evolution of the weight, the strong dependence of consecutive samples typically leads to an underestimation of the variance. This in turn leads to a decrease of the learning rate which is the desired effect of a stationary evolution. In case of a directed evolution of the weight the variance will at least indicate the amount of the current gradient of the evolution despite the strong dependence and thus keep the learning rate high enough to support fast convergence towards the asymptote of the gradient.

An adaptive learning rate such as in Eq. (71) facilitates a spontaneous reorganization of the internal models encoded by the weight vectors of the output neurons 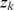 in case that the input distribution 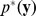 changes (see Fig. S1 in Text S1).

### Details to *Role of the Inhibition*

#### Biased sampling problem

In this section we analyze the influence of the instantaneous output firing rate 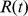 of the learning circuit and derive the analytical result that the output rate 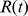 plays the role of a multiplicative weighting of samples during learning. We show how a theoretically optimal inhibition signal can compensate this effect and describe how this compensation is approximated in our experiments.

We start with the assumption that the input signal 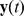 can be described by some stationary stochastic process. An empirical estimate of its stationary distribution can be obtained by measuring the relative duration of presentation of every different discrete value 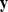 in a time window of length 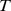. The accuracy of this empirical estimate of the input distribution can be increased by using a longer time window 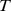, such that in the limit of an infinitely large time window the estimate will converge to the true stationary input distribution of 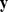, denoted by 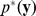:
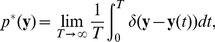
(72)where 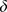 is a vectorized version of the Kronecker Delta with 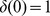 and 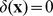, if 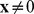.

However, even though the WTA-circuit receives this time-continuous input stream 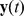, the spike-triggered STDP rule in Eq. (5) and (7) updates the model parameters - i.e. the synaptic weights - only at those time points where one of the output neurons spikes. We denote by 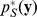 the (empirical) distribution that is obtained from the observations of 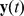 at the first 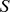 spike events 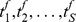:
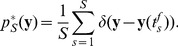
(73)The distribution 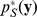 that is seen by the learning rule in Eq. (5) depends not only on the time-continuous input stream 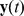, but also on the concrete spike times 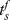 of the circuit. The output spikes thus serve as trigger events at which the continuous input signal is *sampled*.

The spike times 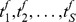 and the total number of spikes 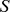 of the whole circuit within a time window of length 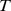 are distributed according to an inhomogeneous Poisson process with the instantaneous rate 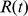. For any stochastic realization of 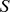 and 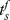 in the time interval 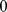 to 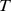, we can derive the expectation of the function 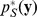 by taking the limit for 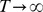 and call this the expected empirical distribution 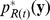. Thus
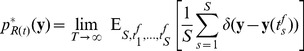
(74)

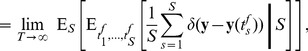
(75)where we divided the expectation into two parts. Firstly we take the expectation over the total number 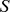 of spikes, secondly we take the expectation over the spike times 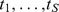, given 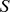. We now make use of the fact that for any inhomogeneous Poisson process 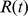, conditioned on the total number of events 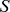 within a certain time window 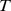, the event times 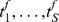 are distributed as order statistics of 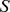 unordered independent samples 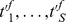 from the probability density 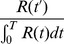. The expectation 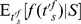 over an arbitrary function 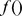 is the integral 
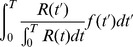
, independent of the event number 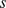, thus
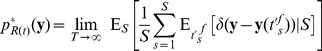
(76)

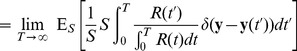
(77)

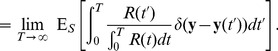
(78)Since the remaining term within the expectation operator 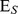 is independent of 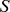 we obtain the final result
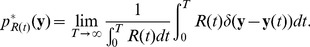
(79)This shows that the output rate 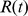 acts as a multiplicative weighting of the contribution of the current input 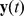 to the expected empirical distribution 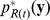, which is learned in the limit of 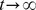 by the simple STDP rule in Eq. (5) and (7).

It turns out that the condition of a constant rate 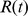 is by far stronger than necessary. In fact, it is easy to see from a comparison of Eq. (72) and Eq. (79), that 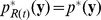 for all values of 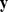 if and only if the relative weight for the input value 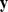, which is 
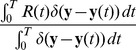
, is independent of 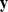 in the limit 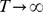. This is certainly true if 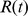 and 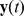 are stochastically independent, i.e. 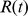 is not correlated to the occurrence of any specific value of 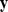.

#### Inhibition model in computer simulations

In our computer simulation the inhibition is implemented by adding a strongly negative impulse to the membrane potential of all 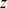-neurons whenever one of them fires, which decays with a time constant of 5 ms back to its resting value. In addition, a noise term 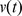 is added to the membrane potential 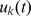 that models background synaptic inputs through an Ornstein-Uhlenbeck (OU) process (as proposed in [110] for modeling in-vivo conditions) and causes stochastic firing. For each experiment, all parameters for the inhibition model are listed in “Simulation Parameters” in the Supplementary Material.

### Details to *Continuous-Time Interpretation with Realistically Shaped EPSPs*

Let the external input vector 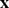 consist of multiple discrete-valued functions in time 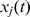, and let us assume that for every input 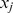 there exists an independent Poisson sampling process with rate 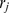 which generates spike times for the group of neurons 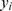 with 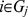. At every spike time 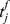 there is exactly one neuron in the group that fires a spike, and this is the neuron that is associated with the value 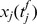. First, we analyze additive step-function EPSPs, i.e. the postsynaptic activation 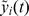 is given by the convolution in Eq. (17) where 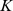 is a step-function kernel with 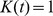 for 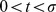 for a fixed EPSP-duration 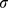 and 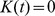 otherwise. In order to understand the resulting distribution 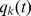 in Eq. (4) as Bayesian inference we extend our underlying generative probabilistic model 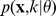 such that it contains multiple instances of the variable vector 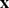, called 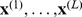, where 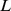 is the total number of spikes from all input neurons 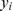 within the time window 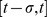. We can see every spike as a single event in continuous time. The full probabilistic model is defined as
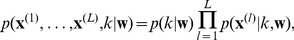
(80)which defines that the multiple instances are modeled as being conditionally independent of each other, given 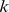. Let the vectors 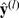 describe the corresponding spike “patterns” in which every binary vector 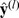 has exactly one 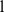 entry 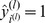. All other values are zero, thus it represents exactly one evidence for 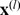, i.e. 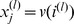, with 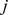, s.t. 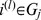, according to the decoding in Eq. (8).

Due to the conditional independences in the probabilistic model every such evidence, i.e. every spike, contributes one factor 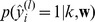 to the likelihood term in the inference of the hidden node 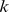. The inference is expressed as 
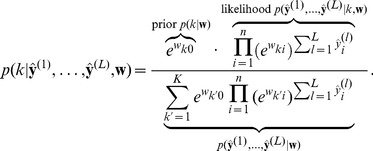
(81)The identity 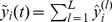 reveals that the above posterior distribution is realized by the relative spike probability 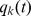 of the network model according to Eq. (4), where 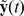 replaces 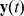 in the computation of the membrane potential 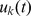. Due to the step function 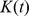 the result of the convolution in 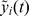 equals the number of spikes within the time window 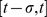 from neuron 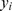. The factor 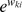, which has the meaning 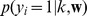 in the network model, is multiplied 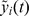 times to the likelihood.

The above discrete probabilistic model gives an interpretation only for integer values of 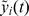, i.e. for functions 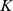 such that 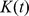 is 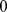 or any positive integer at any time 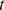. For an interpretation of arbitrarily shaped EPSPs 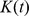 - especially for continuously decaying functions - in the context of our probabilistic model, we now extend this weighting mechanism from integer valued weights to real valued weights by a linear interpolation of the likelihood in the log-space.

The obvious restrictions on the EPSP function 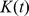 are that it is non-negative, zero for 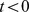, and 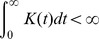, in order to avoid acausal or nondecaying behavior, and unboundedly growing postsynaptic potentials at constant input rates. We assume the normalization 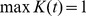. Let again 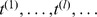 be the times of the past spiking events and 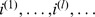 be the indices of the corresponding input neurons. The output distribution 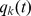
can be written as
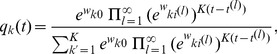
(82)which nicely illustrates that every single past spike at time 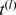 is seen as an evidence in the inference, but that evidence is weighted with a value 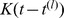, which is between 0 and 1.

The analogous interpolation for continuous-valued input activations 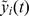 yields the learning rule in Eq. (18), which is illustrated in Fig. 2 as the “Complex STDP rule” (blue dashed curve). The resulting shape of the LTP part of the STDP curve is determined by the EPSP shape defined by 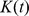. The positive part of the update in Eq. (18) is weighted by the value of 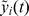 at the time of firing the postsynaptic spike. Negative updates are performed if 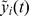 is close to zero, which indicates that no presynaptic spikes were observed recently.

The proof of stochastic convergence does not explicitly assume that 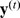 is a binary vector, but is valid for any (positive) random variable vector 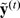 with finite variance. Further, the proof assumes the condition that in every group 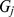 the sum of the input activities 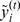 is 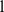 at all times or at least at those points in time at which one 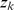 neuron of the WTA-circuit fires. The condition can be relaxed such that the sum per group does not have to be equal to 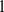 but to any arbitrary (positive) constant if the corresponding normalization constraint is adapted accordingly. Due to the decaying character of the EPSP shape, this sum will never stay constant, even for very regular input patterns. If we only assumed a constant average activation within a group, allowing for stochastic fluctuations around the target value, it turns out that this condition alone is not enough. We need to further assume that these stochastic fluctuations in the sum of every input group 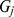 are stochastically independent of the circuit's response 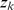. This assumption is intricate and may depend on the data and the learning progress itself, so it will usually not be exactly fulfilled. We can, however, argue that we are close to independence if at least the sum of activity in every group 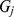 is independent of the value of the underlying abstract variable 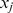.

In our simulations we obtain the input activations 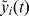 by simulating biologically realistic EPSPs at every synapse, using 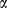-kernels with plausible time constants to model the contributions of single input spikes.

### Details to *Spike-timing dependent LTD*

**Figure 9:**
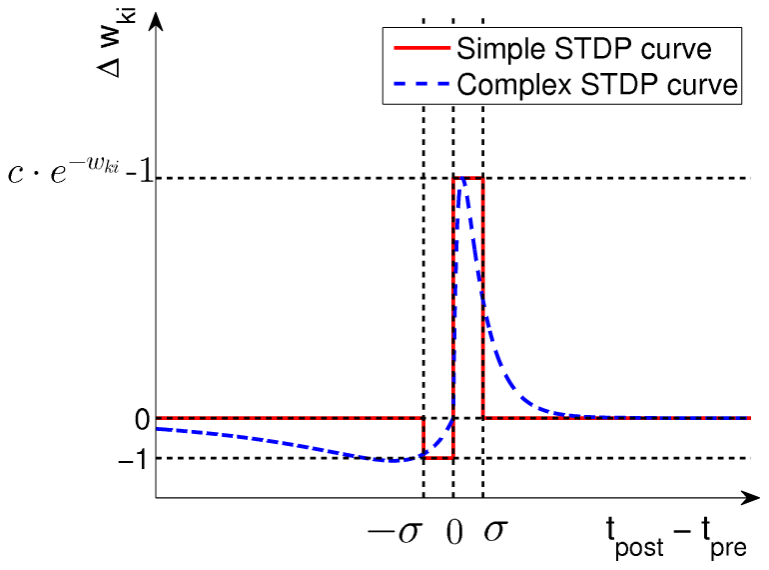
STDP learning curves with time-dependent LTD. Under the simple STDP model (red curve), weight-dependent LTP occurs only if the postsynaptic spike falls within a time window of length 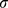 after the presynaptic spike, and LTD occurs in a time window of the same length, but for the opposite order of spikes. This can be extended to a more complex STDP rule (blue dashed curve), in which both LTP and LTD follow 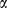-kernels with different time constants, typically with longer time-constants for LTD.

We formalize the presynaptic activity of neuron 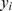 *after* a postsynaptic spike at time 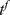 by 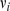, s.t. 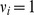 if there is a spike from neuron 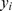 within the time window 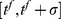 and 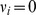 otherwise. This trace is used purely for mathematical analysis, and cannot be known to the postsynaptic neuron at time 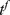, since the future input activity is unknown. Mechanistically, however, 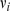 can be implemented as a trace updated by postsynaptic firing, and utilized for plasticity at the time of presynaptic firing [113]. Let us now consider the STDP rule illustrated by the red curve in Fig. 9, where a depression of the synapse happens only if there is a presynaptic spike within the short time window of length 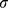 *after* the postsynaptic spike, i.e. if 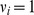. The application of this STDP-rule in our neuronal circuit is equivalent to the circuit-spike triggered update rule
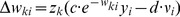
(83)which replaces Eq. (5). In analogy to Eq. (6) the equilibrium of this new update rule can be derived as
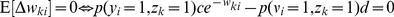
(84)

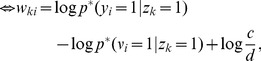
(85)under the assumption that 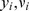 and 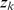 are sampled from a stationary distribution 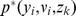. This shows that the synaptic weights can be interpreted as the log-likelihood ratio of the presynaptic neuron firing before instead of after the postsynaptic neuron. In other words, the neuron's synaptic weights learn the contrast between the current input pattern 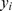 that caused firing, and the following pattern of activity 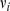. Note that any factor 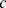 (for LTP) or 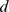 (for LTD) only leads to a constant offset of the weight which - under the assumption that the offset is the same for all synapses - can be neglected due to the WTA circuit (see Methods “Weight offsets and positive weights”).

Similarly to our analysis for the standard SEM rule, we can derive a continuous-time interpretation of the timing-dependent LTD rule. As we did in Eq. (17), we can define
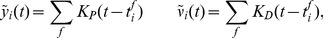
(86)where 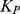 is the same convolution kernel as in Eq. (17), and 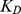 is an arbitrary but time-inversed kernel, such that 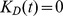 for positive 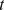 and 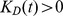 for negative 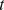. The value of 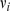 thus reflects a time-discounted sum of presynaptic activity immediately after the postsynaptic spike.

The complex STDP rule from Fig. 2, which models LTD as a constant time-independent depression, can be seen as an extreme case of the spike-timing dependent LTD rule. If 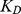 is a step function with 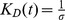 in the interval 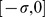 and 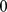 everywhere else, then 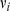 is just the average rate of presynaptic activity in the time interval 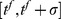 following a postsynaptic spike. In the limit of 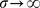 this is equivalent to the overall spiking rate of the neuron 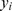, which is proportional to the marginal 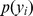 in the probabilistic model. Precisely, 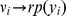, where 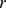 is the base firing rate of an active input in our input encoding model. The equilibrium point of every weight 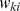 becomes 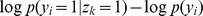, neglecting the offsets induced by the constants 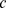,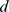 and 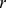. It is easy to see that the probabilistic interpretation of the neuronal model from Eq. (4) is invariant under the transformation 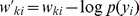, since
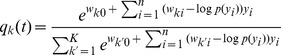
(87)

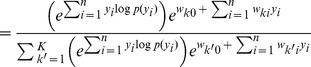
(88)

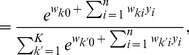
(89)which proves that in our network model the complex STDP rule from Fig. 2 is equivalent to an offset-free STDP rule in the limit of an arbitrarily long window for LTD. In practice, of course, we can assume that the times between pre- and post-synaptic spikes are finite, and we have shown in Fig. 4 that as a result, very realistic shapes of STDP curves emerge at intermediate stimulation frequencies.

## Supporting Information

Text S1 **Supplement.** Derivation of Variance tracking, Adaptation to changing input distributions, Invariance to Time-Warping, Simulation Parameters.
(PDF)
