# Supplementary material for: Bayesian Computation Emerges in Generic Cortical Microcircuits through Spike-Timing-Dependent Plasticity
Source: PLoS Comput Biol. 2013 Apr 25;9(4):e1003037. doi: 10.1371/journal.pcbi.1003037 (PMC3636028; doi:10.1371/journal.pcbi.1003037)
Supplement: Text S1 — Supplement. Derivation of Variance tracking, Adaptation to changing input distributions, Invariance to Time-Warping, Simulation Parameters. (PDF) [file pcbi.1003037.s001.pdf]

Supplement

Derivation of Variance tracking

For the derivation of Eq. (70), let q be a random variable distributed according to a Beta-Distribution with parameters a and b

$$p(q) = \frac{\Gamma(a+b)}{\Gamma(a)\Gamma(b)} p^{a-1}(1-p)^{b-1} \quad . \tag{S.1}$$

Let $w = \log q$, than w is distributed as follows:

$$p(w) = \frac{\Gamma(a+b)}{\Gamma(a)\Gamma(b)} (e^w)^a (1-e^w)^{b-1} \tag{S.2}$$

In order to calculate $\mathrm{E}[w]$ and $\mathrm{E}[w^2]$ we use the moment-generating function of $p(w)$

$$M_w(s) = \int_{-\infty}^{0} e^{sw} p(w) \mathrm{d}w = \tag{S.3}$$

$$= \frac{\Gamma(a+b)}{\Gamma(a)\Gamma(b)} \int_{-\infty}^{0} (e^w)^{a+s} (1-e^w)^{b-1} \mathrm{d}w = \tag{S.4}$$

$$= \frac{\Gamma(a+b)}{\Gamma(a)\Gamma(b)} \frac{\Gamma(a+s)\Gamma(b)}{\Gamma(a+b+s)} = \frac{\Gamma(a+b)\Gamma(a+s)}{\Gamma(a)\Gamma(a+b+s)} \quad . \tag{S.5}$$

The first and the second derivative of M_w read

$$M_w'(s) = \frac{\Gamma(a+b)\Gamma(a+s)}{\Gamma(a)\Gamma(a+b+s)} (\psi(a+s) - \psi(a+b)) \tag{S.6}$$

$$M_w''(s) = \frac{\Gamma(a+b)\Gamma(a+s)}{\Gamma(a)\Gamma(a+b+s)} \Big((\psi(a+s) - \psi(a+b))^2 + \psi_1(a+s) - \psi_1(a+b+s) \Big) \quad . \tag{S.7}$$

Since $\mathrm{E}[w] = M_w'(0)$ and $\mathrm{E}[w^2] = M_w''(0)$ we get

$$\mathrm{E}[w] = \psi(a) - \psi(a+b)$$

$$\mathrm{E}[w^2] = \mathrm{E}[w]^2 + \psi_1(a) - \psi_1(a+b)$$

which can be simplified using the approximations $\psi(x) \approx \log(x)$ and $\psi_1(x) \approx \frac{1}{x}$ to

$$\mathrm{E}[w] \approx \log \frac{a}{a+b} \qquad \mathrm{E}[w^2] \approx \frac{1}{a} + \frac{1}{a+b} \tag{S.8}$$

Adaptation to changing input distributions

In this computer experiment, 10 output neurons learned implicit generative models for images of handwritten digits from the MNIST database. The same procedure for encoding the images by spike trains as in Fig. 6 was used. Initially, only images representing the digits 0 and 3 were presented, and the WTA circuit learned accurate probabilistic models for these images. After 100 seconds of learning, the input distribution was changed, and a third class of inputs, images of handwritten digits 4, was introduced. Through the adaptive learning rate from Eq. (71), the z_k neurons spontaneously reorganized, and two output neurons changed their internal models to represent the new digit 4. In the end, an accurate generative model for all three types of input images was learned.

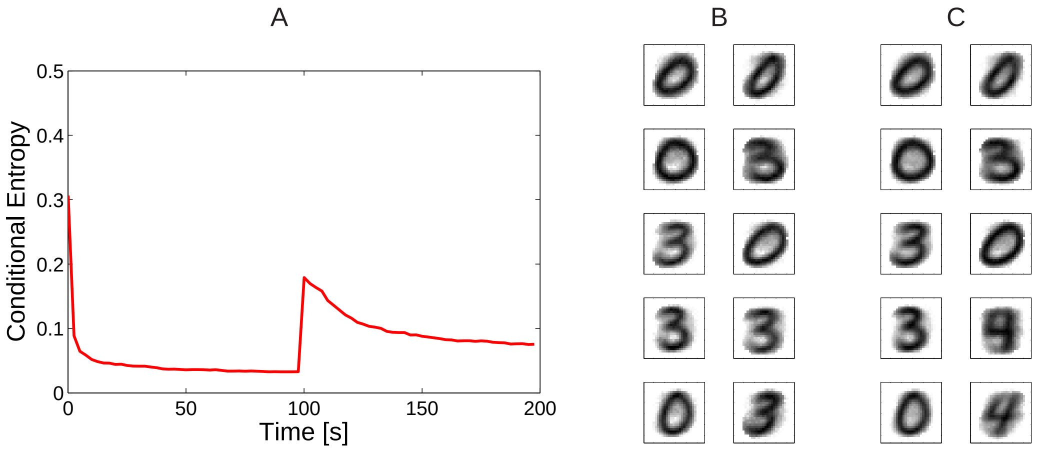

Figure S1: **Spontaneous reorganization of the ensemble of internal models when the input distribution $p^*(\mathbf{y})$ changes.** **A**: Time course of conditional entropy when after 100 s new, previously unseen samples of images of handwritten digits 4 were added to samples of handwritten digits 0 and 3. **B**: Weight vectors of the 10 output neurons after 100 s of learning (before the change of the input distribution). **C**: Spontaneous reorganization of these weight vectors after further 100 s. The weight vectors of two output neurons z_k have developed internal models for two ways of writing the (new) digit 4. Encoding of handwritten digits from MNIST by spike trains $\mathbf{y}$ is as in Fig. 6. The adaptive learning rate in Eq. (71) was used for this experiment.

Invariance to Time-Warping

Simulation Parameters

All simulations were carried out in MATLAB, with a simulation time step of 1 ms. The time constant of the OU process that modeled background synaptic inputs was set to 5 ms, its variance to 2500.

Simulations for Fig. 3:

Input generation:

For each input image pixels were drawn over a 28 x 28 array from one of 4 symmetrical Gaussians with $\sigma^2 = 10$ and centers at (14,8), (16,22), (9,15), (20,14), with maximal probability 0.3 for any pixel to be drawn (causing high variability of samples from the same Gaussian). In addition any pixel was drawn with probability 0.03 (added noise).

When an output neuron z_k fired, on average only 8.6$\%$ of the input neurons y_i had fired during the preceding 10 ms (the time window for potentiation according to the STDP rule in Eq. (5). Hence for over 90$\%$ of the pixels no spike was received within that time window from either one of the two neurons y_i that encoded the value of this pixel by population coding. The corresponding average activity level of all input synapses was at 0.182.

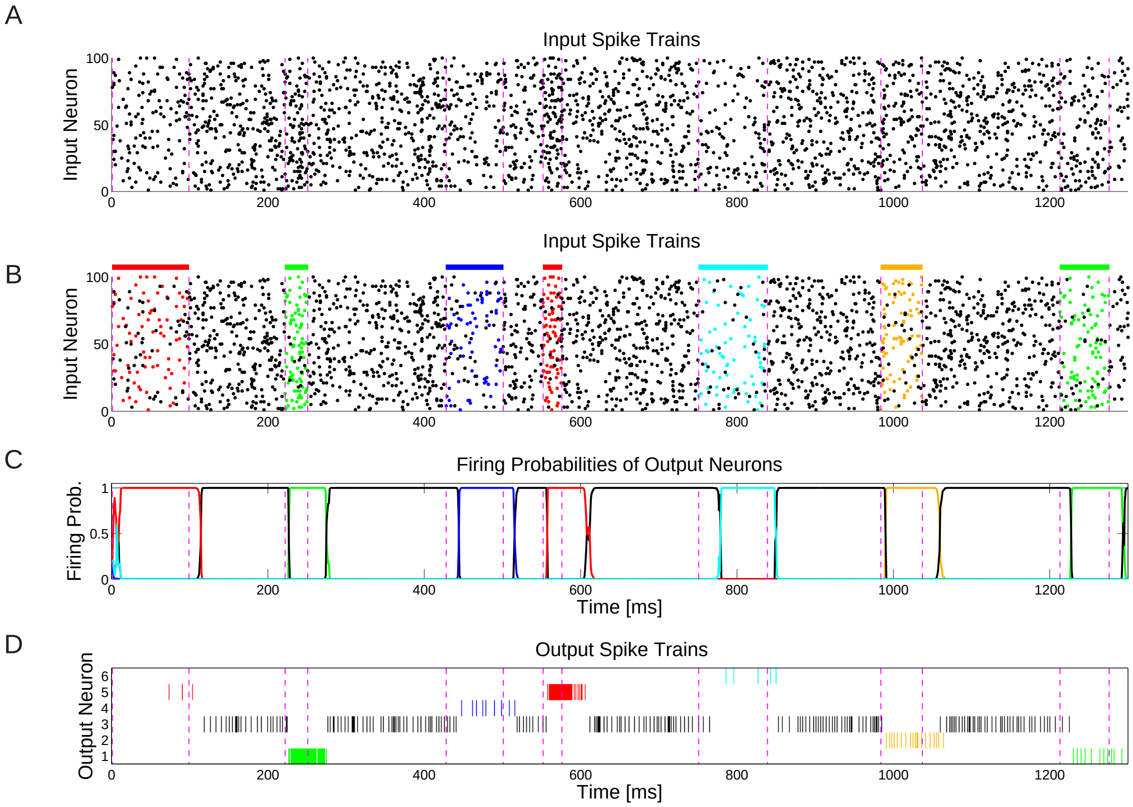

Figure S2: **Generalization capability of the output neurons from Fig. 7 for time-warped variation of the input patterns. A, B**: Another test input presented to the circuit from Fig. 7. The noise-embedded spike patterns are now compressed or stretched from 50 ms to a random length between 25 and 100 ms. Such time-warped versions of these patterns had never been presented during learning via STDP. **C, D**: Firing probabilities and spike outputs of the same 6 output neurons as in Fig. 7. They demonstrate that the emergent discrimination ability of these 6 output neurons automatically generalizes to time-warped input patterns (embedded into noise).

In the variation with superimposed background oscillations at 20 Hz the firing rates of input neurons y_i did not rise, but the average synaptic activity level at the time of an output spike rose to 0.215, an increase of around 18%. This leads to an increased learning rate.

The mean (offset) μ_{ou} of the OU-noise was set to 200, the initial value A_{inh} of lateral inhibition (caused by a firing of a z-neuron) was set to 3000, its resting value O_{inh} to 550. For the version with background oscillations (at 20 Hz) the amplitude of the oscillation was set to 500 (mean = 0), and the phase was shifted by 5 ms for the z-neurons, $A_{inh} = 3000$, $O_{inh} = 650$.

Simulation for Fig. 5:

$\mu_{ou} = 1000, A_{inh} = 3000, O_{inh} = 550.$

Simulation for Fig. 4:

In Figs. 4 A-C pre- and post-synaptic neurons were forced to fire at frequencies of $1, 20,$ and 40 Hz with different time delays. The weight was kept fixed at $w = 3.5$ for $c = e^{-5}$, and the learning rate was kept fixed at $\eta = 0.5$. For Fig. 4D we simulated a pre-synaptic burst consisting of 5 spikes with 20 ms time difference, and a post-synaptic burst of 4 spikes, also with 20 ms time difference. The starting points of these bursts were shifted relative to each other. We kept the weight fixed at $w = 3.5$ for $c = e^{-5}$, and the learning rate fixed at $\eta = 0.1$, and added up the resulting weight changes for all 4 postsynaptic spikes.

Simulation for Fig. 6:

$\mu_{ou} = 250, A_{inh} = 2000, O_{inh} = 400.$

Simulation for Fig. 7 and Suppl. Fig. S2:

$\mu_{ou} = 250, A_{inh} = 1500, O_{inh} = 1000.$

Simulation for Suppl. Fig. S1:

$\mu_{ou} = 250, A_{inh} = 2700, O_{inh} = 400.$
